# Domino ring-opening–ring-closing enyne metathesis vs enyne metathesis of norbornene derivatives with alkynyl side chains. Construction of condensed polycarbocycles

**DOI:** 10.3762/bjoc.14.248

**Published:** 2018-10-25

**Authors:** Ritabrata Datta, Subrata Ghosh

**Affiliations:** 1 School of Chemical Sciences, Indian Association for the Cultivation of Science, Jadavpur, Kolkata 700 032, India

**Keywords:** Diels–Alder reaction, domino process, enyne metathesis, natural products, polycarbocycles

## Abstract

The metathesis of norbornene derivatives with alkynyl side-chain with Grubbs’ ruthenium alkylidine as catalyst has been investigated with the objective of constructing condensed polycyclic structures. This investigation demonstrated that the generally observed domino reaction course involving a ring-opening metathesis of the norbornene unit and a ring-closing enyne metathesis is influenced to a great extent by the nature of the functional group and the substrate structure and may follow a different reaction course than what is usually observed. In cases where ROM–RCEYM occurred, the resulting 1,3-diene reacts in situ with the dienophile to provide condensed tetracyclic systems.

## Introduction

**Scheme 1:**
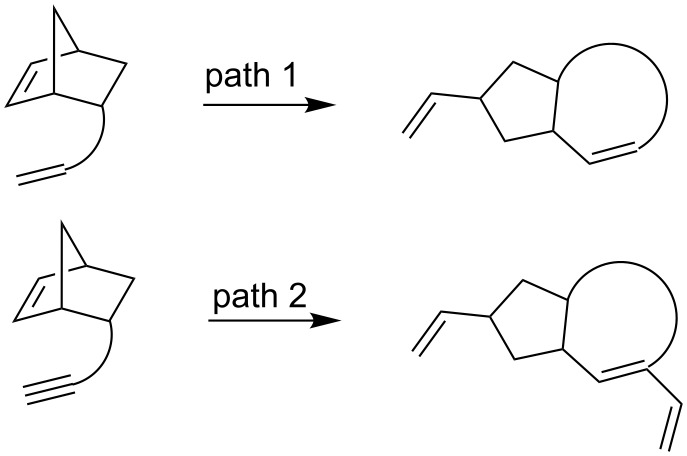
Metathesis of norbornene derivatives.

The metathesis of norbornene derivatives having an alkene side-chain on the norbornene nucleus with Grubbs’ ruthenium catalysts has been extensively investigated. Generally the reaction proceeds through a domino process involving a ring opening of the norbornene nucleus and ring closing with the alkene side chains to produce ring rearrangement products (path 1, Scheme 1) [1–4]. This protocol has been employed by several groups [5–22] as well as by our group [23–33] for the synthesis of a variety of complex ring systems such as condensed, bridged and spirocycles difficult to obtain otherwise. On the contrary, the domino process involving a ring-opening metathesis (ROM) followed by a ring-closing enyne metathesis (RCEYM) [34–37] of norbornene derivatives with a suitably located alkynyl side-chain on the nucleus (path 2, Scheme 1) to form carbocycles has been less explored. The greatest advantage of this protocol lies in its potential in increasing the molecular complexity through Diels–Alder reaction of the resulting ring system. Domino metathesis of oxa- and aza-norbornenes with alkyne side chains [38–40] as well as norbornene derivatives having ether linked alkynes [41–42] in combination with Diels–Alder reaction of the resulting 1,3-dienes have been investigated to construct polycycles with heteroatoms. In spite of the great potential little attention has been paid [43] for exploring its application in the synthesis of complex carbocyclic ring systems, backbones of innumerable natural products.

**Figure 1:**
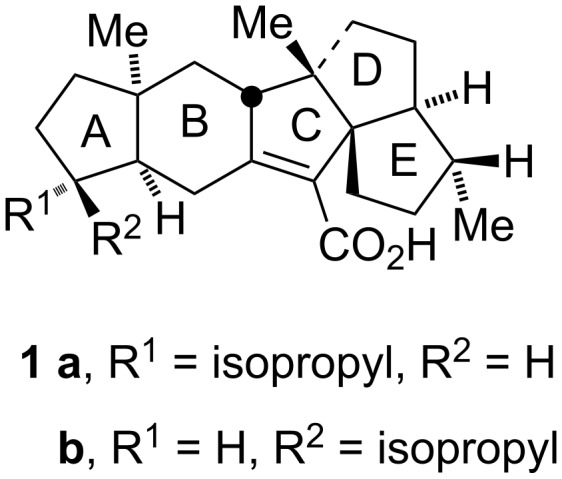
Structures of retigeranic acids A (**1a**) and B (**1b**).

**Scheme 2:**
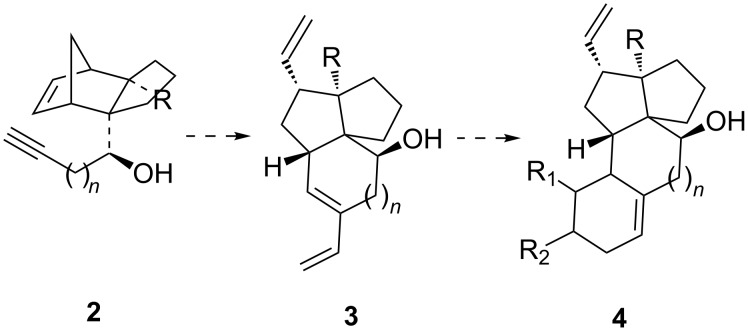
Synthesis plan.

We undertook a program for the synthesis of condensed polycarbocyclic scaffolds using a metathesis of norbornene derivatives with suitably located alkynyl side-chains as the key step. The structurally unique sesterterpenes retigeranic acid A (**1a**) and retigeranic acid B (**1b**, Figure 1) are representative examples of such complex polycyclic structures [44–47]. We speculated that domino ROM–RCEYM of the norbornene derivative **2** would provide the tricyclic 1,3-diene **3** which on Diels–Alder reaction with a dienophile would enable access to condensed polycyclic structures **4** (Scheme 2). Thus an appropriately chosen norbornene derivative and a dienophile may provide the B/C/D/E ring system of retigeranic acids. Herein we describe the results of metathesis of norbornene derivatives **2** with alkynyl side-chains.

## Results and Discussion

**Scheme 3:**
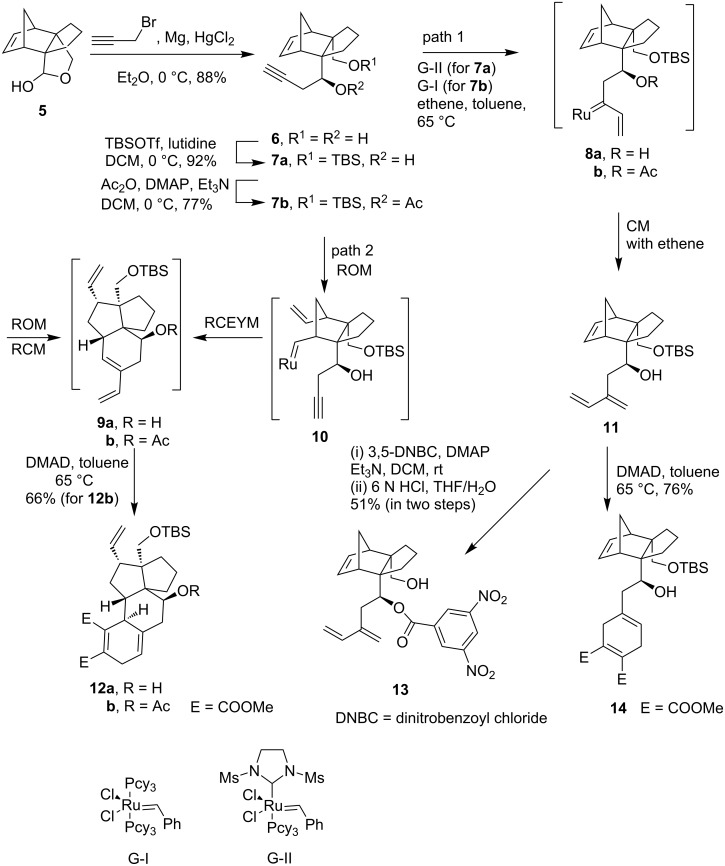
Metathesis of norbornene derivatives **7a** and **7b**.

Initially Grubbs’ 1st generation catalyst (G-I) was used for metathesis of norbornene derivatives **2**. In case G-I failed to accomplish metathesis in the desired direction, 2nd generation catalyst (G-II) was used. The norbornene derivative **7a** was first chosen for investigating ROM–RCEYM. Compound **7a** was prepared in the following way (Scheme 3). Reaction of the known lactol **5** [33] with propargyl magnesium bromide afforded the diol **6** in 88% yield (For detailed experimental procedures and characterization data see Supporting Information File 1). The stereochemical orientation of the secondary hydroxy group was determined through X-ray crystal structure of a compound derived from it in a subsequent step. The primary hydroxy group in the diol **6** was then selectively protected to provide the silyl ether **7a** in 92% yield. Two different paths can be invoked for metathesis of compound **7a**. Metathesis initiation may occur by attack of the ruthenium alkylidene at the alkyne unit to produce the more substituted vinyl alkylidine intermediate **8a** which may undergo concomitant ROM–RCM with the norbornene nucleus to provide the triene **9a** (path 1).

**Figure 2:**
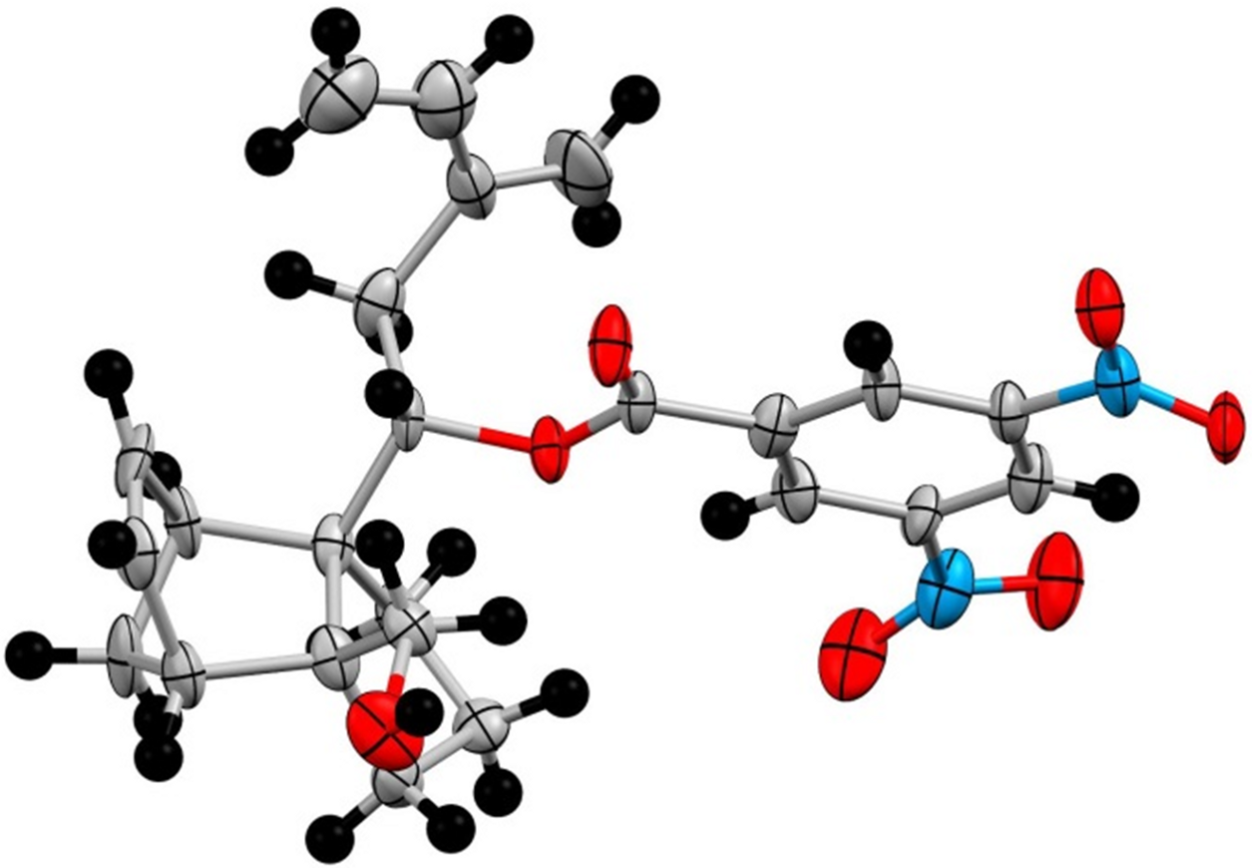
ORTEP of compound **13** (ellipsoids at 30% probability).

Alternatively the metathesis initiation may occur initially at the norbornene double bond to provide the ring-opened ruthenium alkylidine intermediate **10** (path 2). The latter then undergoes RCEYM to provide the tricycle **9a**. With this background a solution of the compound **7a** in toluene under ethylene atmosphere was heated at 65 °C with Grubbs 1st generation catalyst (G-I). Compound **7a** was found to be inert even after a prolonged reaction time. However, with G-II as the catalyst the metathesis went smoothly. Without isolation, the metathesis product was treated in situ with dimethyl acetylenedicarboxylate (DMAD). In case the Diels–Alder reaction would take place through the triene **9a** the tetracyclic structure **12a** would be formed. However, ^13^C NMR spectra of the product revealed the presence of eight methylene carbon signals at δ 28.6, 28.9, 30.9, 33.5, 36.7, 41.1, 45.7 and 68.8, one more aliphatic methylene unit than what the structure **12a** requires (see Supporting Information File 1). This indicates that the metathesis product is not **9a**. The structure of the metathesis product was finally settled by X-ray crystal structure (Figure 2) [48] (see Supporting Information File 2) of the 3,5-dinitrobenzoate derivative **13**, mp 171–172 °C, prepared in two steps (51%) from the metathesis product on reaction with 3,5-dinitrobenzoyl chloride (DNBC) followed by acid-induced desilylation. Thus compound **7b** on metathesis produced exclusively triene **11** and accordingly the structure of the Diels–Alder adduct is **14**. The formation of triene **11** could be attributed to cross metathesis of the ruthenium alkylidene **8a** with ethylene. No product arising out of ROM of norbornene derivative **7a** was formed. It is worth mentioning that Spandl et al. [43] reported the metathesis of norbornene derivatives with an alkynyl side chain affording the major product arising from domino ROM–RCEYM while the enyne metathesis product was observed only in very low yield.

In order to realize our objective and to find out if the free hydroxy group has any influence on the outcome of the metathesis, the hydroxy group in compound **7a** was protected to provide the acetate derivative **7b**. The metathesis of compound **7b** with G-I as the catalyst proceeded smoothly and the resulting product without isolation was allowed to react with DMAD to produce the tetracycle **12b** in overall 66% yield. The structure of compound **12b** was established through analysis of its NMR spectra. Isolation of **12b** dictated that metathesis of **7b** proceeded through the formation of the triene **9b**. Stereochemical assignment to the adduct follows from addition of the dienophile from the least hindered face (opposite to CH_2_OTBS group) of the diene. Thus unlike metathesis of **7a**, metathesis of its acetate analogue **7b** occurred through a domino ROM–RCEYM process. Addition of the Ru-carbene **10** arising from ring opening of norbornene unit in **7b** could add to the acetylenic unit of another molecule of **7b** leading to copolymerization. However, this process generally does not take place under such low molar concentration of the substrate [38–43]. We also did not isolate any copolymerization product. This may be attributed to the much faster rate of addition of the Ru-carbene **10** to the yne unit intramolecularly resulting in ring closure rather than intermolecular addition to an acetylenic unit of another molecule of **7b**. It may be noted that changing the functional group from hydroxy to acetate the metathesis followed a different reaction course.

**Scheme 4:**
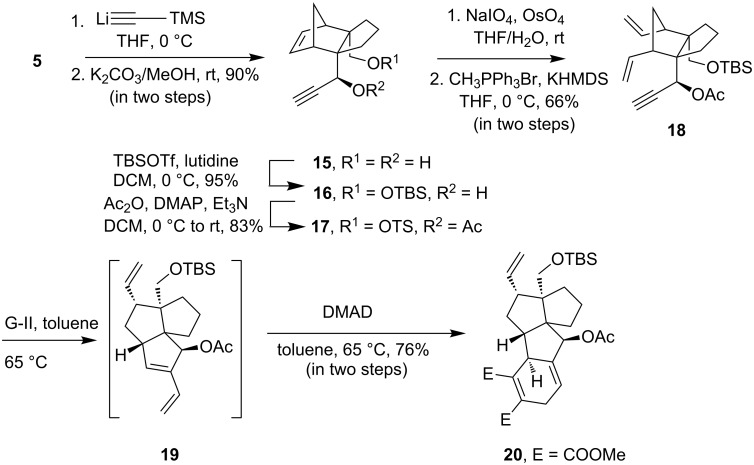
Metathesis of the norbornene derivative **17**.

In order to construct a polycyclic structure analogous to the B/C/D/E ring of retigeranic acids, the norbornene derivative **16** was chosen. Addition of lithium (trimethylsilyl)acetylide to the lactol **5** followed by desilylation by using methanolic K_2_CO_3_ afforded diol **15** (Scheme 4). The primary hydroxy group in compound **15** was selectively protected to produce the silyl ether **16** in 95% yield. The attempted metathesis of compound **16** with G-I or G-II catalyst under the conditions used for the metathesis of **7a** led to a complete recovery of **16**. Since metathesis of the acetate derivative **7b** proceeded smoothly in the desired direction, we chose to use the acetate **17** for metathesis. The acetate **17** also remained inert when subjected to metathesis conditions with G-I as well as with G-II. Neither ring opening of the norbornene nucleus nor cross metathesis of the alkyne with ethylene did occur. To have an understanding about the inertness of **17** towards metathesis we decided to prepare the ring-opened product **18** using an alternative path. The double bond in the norbornene nucleus in compound **17** was cleaved in the traditional way by treatment with OsO_4_/NaIO_4_ and the resulting dialdehyde on Wittig reaction provided the diene **18** in 66% yield in two steps. Amazingly when compound **18 was** treated with G-I or G-II as catalyst, the metathesis was found to take place. After disappearance of the starting material (TLC), the reaction mixture was allowed to react with DMAD. The product obtained in 76% yield was assigned the structure **20** based on spectral data. Isolation of **20** indicates that metathesis of **18** proceeded through RCEYM to produce the triene **19**. The latter then after in situ Diels–Alder reaction with DMAD delivered the product **20**. The tetracyclic compound **20** represents the B/C/D/E tetracyclic core structure of retigeranic acids.

**Figure 3:**
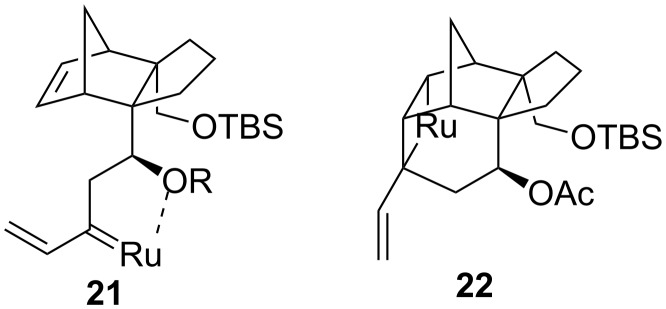
Probable metathesis intermediates.

Based on the above observations a mechanistic rationale regarding the metathesis of norbornene derivatives with an alkynyl side chain may be postulated (Figure 3). Possibly the metathesis is initiated at the acetylenic unit to form the ruthenium alkylidine such as **8**. In case of **8a** the ruthenium alkylidine is stabilized by formation of the chelate **21** (R = H) which prohibits intramolecular addition of the ruthenium alkylidine to form ruthena cyclobutane **22**. The alkylidine **21** then undergoes cross metathesis with ethylene to form the product **11**. The ruthenium alkylidine **8b** possibly fails to form chelate **21** (R = Ac) due to the electron deficient nature of the OAc group. It forms intramolecularly the ruthena cyclobutane **22** which undergoes ring opening to give rise to the triene **9b**. That the metathesis does not proceed through path 2 (Scheme 3) involving ROM–RCM is indicated by failure of the norbornene derivative **17** to undergo ROM. Steric shielding of the acetylenic unit in **17** inhibits metathesis initiation at the acetylenic unit. The norbornene derivative **17** just remains inert under metathesis conditions. Thus metathesis in these examples proceeds through path 1 (Scheme 3).

## Conclusion

In conclusion we have developed a protocol for the synthesis of condensed polycycles from metathesis of norbornene derivatives with alkynyl side-chain. This investigation demonstrated that domino metathesis of norbornene derivatives with alkynyl side-chain requires metathesis initiation at the acetylene unit. Further, the nature of functional groups as well as the substrate structure play a significant role in determining the metathesis reaction course.

## Experimental

General experimental methods are similar as described in [49]

**Synthesis of triene 11.** A solution of the silyl ether **7a** (120 mg, 0.35 mmol) in degassed toluene (7 mL) with Grubbs’ catalyst G-II (30 mg, 0.035 mmol) was heated at 65 °C for 6 h under a positive pressure of ethylene atmosphere. After completion (TLC) of the reaction toluene was removed under vacuo. The residual mass was purified by column chromatography (7% EA/PE) to afford diene **11** (89 mg, 69%) as an oil; ^1^H NMR (500 MHz) δ 6.42 (dd, *J* = 11, 17.5 Hz, 1H), 6.12 (s, 2H), 5.28 (d, *J* = 17.5 Hz, 1H), 5.15 (d, *J* = 29 Hz, 2H), 5.04 (d, *J* = 11 Hz, 1H), 3.98 (s, 1H), 3.63 (d, *J* = 10 Hz, 1H), 3.56 (s, 2H), 2.53 (s, 1H), 2.45–2.42 (m, 1H), 2.36–2.22 (m, 3H), 1.95–1.92 (m, 1H), 1.82–1.74 (m, 3H), 1.47–1.38 (m, 2H), 1.33–1.28 (m, 1H), 0.89 (s, 9H), 0.07 (s, 6H); ^13^C NMR (125 MHz) δ 144.3, 139.8, 136.4, 136.1, 117.0, 113.1, 71.9, 68.6, 63.4, 61.2, 52.2, 52.0, 45.8, 36.8, 36.0, 33.8, 28.8, 26.0 (× 3), 18.4, −5.4, −5.6; HRMS–ESI *m*/*z*: [M + Na]^+^ calcd for C_23_H_38_O_2_SiNa 397.2539; found, 397.2537.

**Diels–Alder reaction of diene 11. Synthesis of adduct 14.** A mixture of the diene **11** (40 mg, 0.11 mmol) and dimethyl acetylenedicarboxylate (0.02 mL, 0.16 mmol) in toluene (5 mL) was heated at 65 °C for 2 h. The solvent was removed under reduced pressure and was purified by column chromatography (12% EA/PE) to afford the Diels–Alder adduct **14** (42 mg, 76%) as an oil; ^1^H NMR (500 MHz) δ 6.12–6.11 (m, 2H), 5.52 (s, 1H), 4.26 (s, 1H), 3.89–3.88 (m, 1H), 3.78 (s, 3H), 3.76 (s, 3H), 3.60–3.52 (m, 3H), 3.05–2.97 (m, 3H), 2.45 (s, 1H), 2.32 (s, 1H), 2.18–2.17 (m, 3H), 1.93–1.89 (m, 1H), 1.83–1.78 (m, 2H), 1.75–1.68 (m, 2H), 1.48–1.41 (m, 1H), 1.39–1.33 (m, 2H), 0.88 (s, 9H), 0.08 (s, 6H); ^13^C NMR (75 MHz) δ 169.0, 168.7, 136.4, 135.8, 133.6, 132.4, 132.2, 117.9, 72.0, 68.8, 63.1, 61.2, 52.3 (× 2), 52.1 (× 2), 45.7, 41.1, 36.8, 33.6, 30.9, 28.9, 28.6, 26.0 (× 3), 18.4, −5.4, −5.6; IR: 2952, 1728, 1471 cm^−1^; HRMS–ESI *m*/*z*: [M + Na]^+^ calcd for C_29_H_44_O_6_SiNa 539.2805; found, 539.2802.

**Synthesis of tetracycle 12b.** A solution of the norbornene derivative **7b** (70 mg, 0.18 mmol) in degassed toluene (6 mL) was heated with Grubbs’ catalyst G-I (15 mg, 0.018 mmol) under ethylene atmosphere at 65 °C for 12 h. After completion (TLC) of the metathesis reaction, dimethyl acetylenedicarboxylate (0.04 mL, 0.27 mmol) was added to the reaction mixture. The reaction mixture was then heated for 12 h till the Diels–Alder reaction of the diene **9b** generated in situ was complete. The solvent was removed under vacuo and the product was purified by column chromatography (15% EA/PE) to afford the tetracycle **12b** (66 mg, 66%) as a colorless oil; ^1^H NMR (300 MHz) δ 5.99–5.87 (m, 1H), 5.63–5.57 (m, 1H), 5.35–5.34 (m, 1H), 4.99–4.94 (m, 2H), 3.74 (s, 3H), 3.73 (s, 3H), 3.58–3.48 (m, 2H), 3.25–3.16 (m, 2H), 3.10–3.07 (m, 1H), 2.85–2.75 (m, 1H), 2.14–2.07 (m, 2H), 2.04 (s, 3H), 2.02–1.85 (m, 2H), 1.69–1.59 (m, 4H), 1.53–1.25 (m, 3H), 0.94 (s, 9H), 0.02 (s, 3H), −0.03 (s, 3H); ^13^C NMR (75 MHz) δ 170.1, 169.3, 168.7, 139.5, 136.9, 134.0, 132.5, 115.9, 115.0, 73.4, 65.1, 60.6, 57.6, 56.2, 53.1, 52.4, 52.1, 40.9, 37.6, 36.2, 34.9, 34.7, 28.2, 26.2 (× 3), 22.1, 21.8, 17.9, −5.8, −6.1; IR: 2950, 1737, 1434, 1249 cm^−1^; HRMS–ESI *m*/*z*: [M + Na]^+^ calcd for C_31_H_46_O_7_SiNa 581.2911; found, 581.2914.

**Synthesis of the tetracycle 20.** The dienyne **18** (100 mg, 0.25 mmol) in degassed anhydrous toluene (7 mL) was treated with Grubbs’ catalyst G-II (22 mg, 0.025 mmol) at 65 °C for 5 h. On completion of the reaction (TLC), dimethyl acetylenedicarboxylate (0.06 mL, 0.37 mmol) was added to the resulting reaction mixture. The mixture was heated at 65 °C for 8 h. Removal of the solvent under vacuo followed by column chromatography (15% EA/PE) afforded the Diels–Alder adduct **20** (102 mg, 76%) as a colorless oil; ^1^H NMR (300 MHz) δ 6.06–6.02 (m, 1H), 6.00–5.94 (m, 1H), 5.24–5.21 (m, 1H), 5.03–4.95 (m, 2H), 3.80 (s, 3H), 3.75 (s, 3H), 3.53 (s, 2H), 3.19–3.11 (m, 1H), 3.09–3.02 (m, 1H), 3.00–2.86 (m, 1H), 2.43–2.34 (m, 1H), 2.14 (s, 3H), 2.08–1.98 (m, 2H), 1.96–1.78 (m, 2H), 1.55–1.50 (m, 2H), 1.46–1.41 (m, 1H), 1.34–1.25 (m, 1H), 1.12–0.99 (m, 1H), 0.91 (s, 9H), 0.04 (s, 3H), 0.02 (s, 3H); ^13^C NMR (75 MHz) δ 170.0, 169.0, 167.5, 140.1, 139.3, 139.2, 129.0, 115.3, 109.8, 74.4, 65.6, 62.3, 58.1, 56.7, 54.7, 52.4, 52.3, 41.9, 40.1, 35.9, 35.6, 27.6, 26.2 (× 3), 22.9, 21.1, 18.2, −5.8, −5.9; IR: 2950, 1731, 1434, 1257 cm^−1^; HRMS–ESI *m*/*z*: [M + Na]^+^ calcd for C_30_H_44_O_7_SiNa 567.2754; found, 567.2756.

## Supporting Information

File 1 Experimental and analytical data.

File 2 Crystallographic information for compound **13**.
