# Supplementary material for: Domino ring-opening–ring-closing enyne metathesis vs enyne metathesis of norbornene derivatives with alkynyl side chains. Construction of condensed polycarbocycles
Source: Beilstein J Org Chem. 2018 Oct 25;14:2708–14. doi: 10.3762/bjoc.14.248 (PMC6204787; doi:10.3762/bjoc.14.248)
Supplement: File 1 — Experimental and analytical data. [file Beilstein_J_Org_Chem-14-2708-s001.pdf]

Supporting Information

for

Domino ring-opening–ring-closing enyne metathesis vs enyne metathesis of norbornene derivatives with alkynyl side chains. Construction of condensed polycarbocycles

Ritabrata Datta and Subrata Ghosh*

Address: School of Chemical Sciences, Indian Association for the Cultivation of Science, Jadavpur, Kolkata 700 032, India

Email: Subrata Ghosh* - ocsg@iacs.res.in

*Corresponding author

Experimental and analytical data

Experimental section

General experimental methods are similar to [49].

Synthesis of diol 6. A solution of the known lactol **5** (1.1 g, 5.72 mmol) in anhydrous Et_2O (20 mL) was slowly added to a solution of propargyl magnesium bromide [prepared by adding propargyl bromide (1.97 mL, 22.89 mmol) to a mixture of magnesium turnings (550 mg, 22.88 mmol) and $HgCl_2$ (78 mg, 0.286 mmol) in Et_2O (20 mL)] at 0 °C for 30 min. The reaction was quenched by adding saturated aqueous solution of NH_4Cl (5 mL) and was filtered through celite. The celite was washed by Et_2O (3 × 30 mL). The combined filtrate was washed with brine (5 mL) and dried. The residue after evaporation of organic layer was purified by column chromatography (30% EA/PE) to provide the diol **6** (1.17 g, 88%) as an oil. ^{1}H NMR (300 MHz) δ 6.13-6.06 (2H, m), 4.02 (2H, brs), 3.58 (1H, q, *J* = 4.5 Hz), 3.51-3.43 (2H, m), 2.45-2.33 (4H, m), 2.11-2.01 (3H, m), 1.81-1.64 (3H, m), 1.49-1.35 (2H, m), 1.28-1.17 (1H, m); ^{13}C NMR (75 MHz) δ 137.0, 135.0, 81.9, 73.4, 70.9, 67.4, 61.8, 61.1, 52.0, 51.7, 45.6, 36.5, 33.0, 28.7, 24.8; HRMS (ESI) m/z Calcd for $C_{15}H_{20}O_2Na$ $[M + Na]^+$ 255.1361, found 255.1363.

Synthesis of silyl ether 7a. In a similar manner to a procedure from [49] the diol **6** (1 g, 4.30 mmol) was silylated to afford the silyl ether **7a** (oil, 1.37 g, 92%) as an oil; ^{1}H NMR (300 MHz) δ 6.17 (1H, dd, *J* = 2.7, 5.7 Hz), 6.08 (1H, dd, *J* = 3, 5.7 Hz), 4.36 (1H, s), 3.58-3.49 (3H, m), 2.44-2.31 (4H, m), 2.21-2.14 (1H, m), 1.99 (1H, t, *J* = 2.7 Hz), 1.93-1.87 (1H, m), 1.85-1.64 (3H, m), 1.47-1.35 (2H, m), 1.26-1.16 (1H, m), 0.87 (9H, s), 0.07 (6H, s); ^{13}C NMR (75 MHz) δ 136.2 (X 2), 83.0, 73.1, 69.3, 68.5, 62.9, 61.1, 52.0, 51.9, 45.8, 36.7, 33.2, 28.7, 25.9 (X3), 24.3, 18.3, -5.5, -5.6; HRMS (ESI) m/z Calcd for $C_{21}H_{34}O_2SiNa$ $[M + Na]^+$ 369.2226, found 369.2223.

Synthesis of norbornene derivative 7b. To a solution of the silyl ether **7a** (1.35 g, 3.90 mmol) in anhydrous DCM (15 mL) was added Et_3N (4.31 mL, 31.16 mmol), catalytic amount of 4-dimethylaminopyridine (DMAP) (10 mg) and acetic anhydride (1.47 mL, 15.58 mmol). The mixture was stirred for 72 h. On dilution with water (10 mL) the reaction mixture was extracted with Et_2O (3 × 50 mL). The combined extract was washed with brine and dried. The residue left after evaporation of solvent under vacuo was purified by column chromatography (10% EA/PE) to afford the acetate derivative **7b** as a colorless oil (1.17 g, 77%); IR: 2954, 1743, 1471, 1245 cm^{-1}; ^{1}H NMR (300 MHz) δ 6.26 (1H, dd, *J* = 3, 6 Hz), 6.17 (1H, dd, *J* = 3, 6 Hz), 4.73-4.70 (1H, m), 3.16-3.08 (2H, m), 2.74 (1H, s), 2.56-2.51 (1H, m), 2.50-2.43 (2H, m), 2.28-2.21 (1H, m), 2.01 (3H, m), 1.98-1.95 (1H, m), 1.83-1.67 (3H, m), 1.47-1.25 (4H, m), 0.88 (9H, s), -0.01 (3H, s), -0.02 (3H, s); ^{13}C NMR (75 MHz) δ 170.0, 138.9, 135.1, 80.6, 74.4, 70.7, 67.0, 61.6, 61.1, 51.2, 49.4, 44.6, 36.6, 34.6, 27.8, 26.0 (X 3), 23.4, 21.3, 18.4, -5.4, -5.5; HRMS (ESI) m/z Calcd for $C_{23}H_{36}O_3SiNa$ [M + Na]$^+$ 411.2332, found 411.2330.

Synthesis of dinitrobenzoate derivative 13. A solution of the diene **11** (200 mg, 0.53 mmol) in DCM (10 mL) was treated with Et_3N (0.6 mL, 4.27 mmol), catalytic amount of DMAP (10 mg) and 3,5-dinitrobenzoyl chloride (493 mg, 2.14 mmol) was stirred at rt for 72 h. A saturated aqueous solution of $NaHCO_3$ (5 mL) was added to the reaction mixture. The reaction mixture was extracted with Et_2O (3 × 20 mL). The combined organic extract was washed with brine (5 mL) and dried. Evaporation of solvent under vacuo provided a viscous mass (178 mg). Without any purification and characterization this product was directly used for the next step. To a solution of this mass in THF (10 mL) was treated with 6 N HCl (20 mL) at rt for 3 h. The reaction mixture was extracted with diethyl ether (3 × 20 mL). The combined ether layer was washed with saturated $NaHCO_3$ (5 mL), brine (5 mL) and dried.

Evaporation of solvent under vacuo followed by column chromatography (20% EA/PE) of the residual mass provided the hydroxy-compound **13** (122mg, 51%) as a white solid which was crystallized (20% PE/DCM) to afford the pure compound **13**, m.p. 171-172 °C; IR: 2954, 1735, 1627, 1548 cm^{-1}; ^1H NMR (300 MHz) δ 9.19 (1H, t, *J* = 2.1 Hz), 9.03-9.02 (2H, m), 6.39 (1H, dd, *J* = 3, 6 Hz), 6.35-6.34 (1H, m), 6.30-6.24 (1H, m), 5.34-5.28 (1H, m), 5.20-5.09 (2H, m), 4.94 (2H, d, *J* = 6 Hz), 3.38 (1H, d, *J* = 10.2 Hz), 3.15 (1H, d, *J* = 10.2 Hz), 2.80-2.75 (1H, m), 2.73-2.46 (4H, m), 2.06-1.28 (7H, m), 0.97-0.85 (1H, m); ^{13}C NMR (75 MHz) δ 161.5, 148.8, 142.4 (X 2), 138.6, 138.5, 135.2, 134.8, 129.3 (X 2), 122.2, 119.9, 114.0, 78.5, 66.9, 62.8, 61.0, 51.9, 50.2, 45.3, 36.3, 35.9, 34.6, 28.3; HRMS (ESI) m/z Calcd for $C_{24}H_{27}N_2O_7$ $[M + H]^+$ 455.1818, found 455.1816.

Synthesis of diol 15. In a similar manner to a procedure from [49] the lactol **5** (1.0 g, 5.20 mmol) in THF (20 mL) was added to a solution of lithium (trimethylsilyl) acetylide The crude product obtained as above was dissolved in dry MeOH (15 mL) and then K_2CO_3 (1.44 g, 10.40 mmol) was added to it. The mixture was stirred for 2 h and was filtered through celite. The celite bed was washed with Et_2O (20 mL). Then the filtrate was evaporated under vacuo and the residue was purified by column chromatography (30% EA/PE) to afford the diol **15** (1.02 g, 90%) as a liquid; ^1H NMR (300 MHz) δ 6.18 (1H, s), 6.12 (1H, s), 4.86 (1H, brs), 4.22 (1H, brs), 4.16 (1H, s), 3.46 (2H, dd, *J* = 11.4, 35.7 Hz), 2.67 (1H, s), 2.42 (1H, s), 2.36 (1H, s), 2.31-2.25 (1H, m), 2.07-2.01 (1H, m), 1.83-1.76 (2H, m), 1.74-1.61 (1H, m), 1.54-1.37 (3H, m); ^{13}C NMR (75 MHz) δ 136.6, 136.2, 84.3, 72.9, 67.7, 67.4, 63.0, 60.0, 52.9, 52.3, 45.5, 36.7, 34.6, 28.6; HRMS $C_{14}H_{19}O_2$ $[M + H]^+$ 219.1385, found 219.1386.

Synthesis of silyl ether 16. In a similar manner to a procedure from [49] the diol **15** (995 mg, 4.56 mmol) was converted to the silyl ether **16** as colorless oil (1.44 g, 95%); ^1H NMR (300

MHz) δ 6.19 (1H, s), 6.07-6.06 (1H, m), 4.70 (1H, s), 4.15 (1H, s), 3.55 (1H, d, J = 10.5, Hz), 3.41 (1H, d, J = 10.5 Hz), 2.66 (1H, s), 2.37-2.32 (3H, m), 1.91-1.63 (4H, m), 1.50-1.32 (3H, m), 0.84 (9H, s), 0.04 (3H, s), 0.03 (3H, s). ^{13}C NMR (75 MHz) δ 136.6, 135.9, 84.3, 71.9, 68.7, 67.3, 63.3, 60.0, 52.9, 52.2, 45.6, 36.9, 34.6, 28.6, 25.8 (X 3), 18.1, -5.6, -5.7; HRMS: $C_{20}H_{32}O_2SiNa$ [M + Na]$^+$ 355.2069, found 355.2068.

Synthesis of acetate 17. Following the procedure described for synthesis of the acetyl derivative **7b**, the carbinol **16** (800 mg, 2.41 mmol) was converted to the norbornene derivative **17** as colorless oil (739 mg, 83%) using acetic anhydride (0.91 mL, 9.62 mmol) and Et_3N (2.66 mL, 19.24 mmol) after purification by column chromatography (10% EA/PE). The acetate displayed the following spectral data: IR: 2955, 1747, 1469, 1228 cm^{-1}; ^{1}H NMR (300 MHz) δ 6.22-6.21 (2H, m), 4.85 (1H, d, J = 1.8 Hz), 3.18 (1H, d, J = 8.7 Hz), 2.98 (1H, dd, J = 9, 1.5 Hz), 2.72 (2H, dd, J = 11.1, 1.2 Hz), 2.39 (1H, d, J = 2.1 Hz), 2.29-2.27 (1H, m), 2.01 (3H, s), 1.98-1.92 (1H, m), 1.82-1.68 (3H, m), 1.56-1.23 (3H, m), 0.86 (9H, s), -0.04 (6H, s); ^{13}C NMR (75 MHz) δ 169.5, 138.4, 135.6, 81.2, 73.2, 69.0, 66.2, 61.5, 60.1, 51.9, 49.8, 44.4, 36.5, 36.0, 27.5, 25.9 (X 3), 21.0, 18.3, -5.4, -5.6; HRMS: $C_{22}H_{35}O_3Si$ [M + H]$^+$ 375.2355, found 375.2351.

Synthesis of the dienyne 18. In a similar manner to a procedure from [49] the norbornene derivative **17** (600 mg, 1.60 mmol) was subjected to cleavage to afford the corresponding bis-aldehyde. A solution of methyl triphenyl phosphonium bromide (2.29 g, 6.41 mmol) in THF (20 mL) was treated with KHMDS [0.5 (M) in toluene] (16.0 mL, 8.01 mmol) at 0 °C for 30 min. To it a solution of the bis-aldehyde obtained above in THF (10 mL) was added at 0 °C for 1 h. The reaction was quenched by saturated aqueous NH_4Cl (5 mL). The resulting mixture was extracted with Et_2O (3 × 30 mL). The combined ether layer was washed with brine (5 mL) and dried. Solvent was evaporated under reduced pressure and purified by

column chromatography (8% EA/PE) to afford the dienyne **18** (420 mg, 66%) as an oil; IR: 2954, 1743, 1471, 1238 cm^{-1}; ^{1}H NMR (400 MHz) δ 6.24-6.11 (1H, m), 5.90-5.87 (1H, m), 5.76 (1H, s), 5.01-4.93 (4H, m), 4.03 (1H, d, J = 10.4 Hz), 3.52 (1H, d, J = 10.4 Hz), 2.47 (1H, d, J = 2.4 Hz), 2.34-2.26 (2H, m), 2.02 (3H, s), 1.77-1.61 (7H, m), 1.54-1.51 (1H, m), 0.90 (9H, s), 0.06 (3H, s), 0.03 (3H, s); ^{13}C NMR (100 MHz) δ 169.5, 139.3, 139.1, 115.0, 114.7, 82.1, 75.2, 67.2, 64.2, 60.6, 60.5, 54.1, 53.2, 38.9, 37.6, 36.4, 26.0 (X 3), 21.8, 21.1, 18.4, -5.5, -5.6; HRMS (ESI) m/z Calcd for $C_{24}H_{38}O_3SiNa$ $[M + Na]^+$ 425.2488, found 425.2487.

Crystal data for compound **13**

Compound reference	**13**
Chemical formula	$C_{24}H_{26}N_2O_7$
Formula Mass	454.47
Crystal system	Triclinic
a/Å	8.25(2)
b/Å	10.46(2)
c/Å	13.93(3)
α/°	77.91(4)
β/°	76.47(4)
γ/°	85.92(4)
Unit cell volume/$Å^3$	1142(5)
Temperature/K	293(2)
Space group	*P*1,¯
No. of formula units per unit cell, *Z*	2
Radiation type	MoKα
Absorption coefficient, μ/mm^{-1}	0.098
No. of reflections measured	3415
No. of independent reflections	2381
R_{int}	0.0399
Final R_1 values ($I > 2\sigma(I)$)	0.1576
Final $wR(F^2)$ values ($I > 2\sigma(I)$)	0.4564
Final R_1 values (all data)	0.1863
Final $wR(F^2)$ values (all data)	0.4746
Goodness of fit on F^2	1.845
CCDC number	1847091

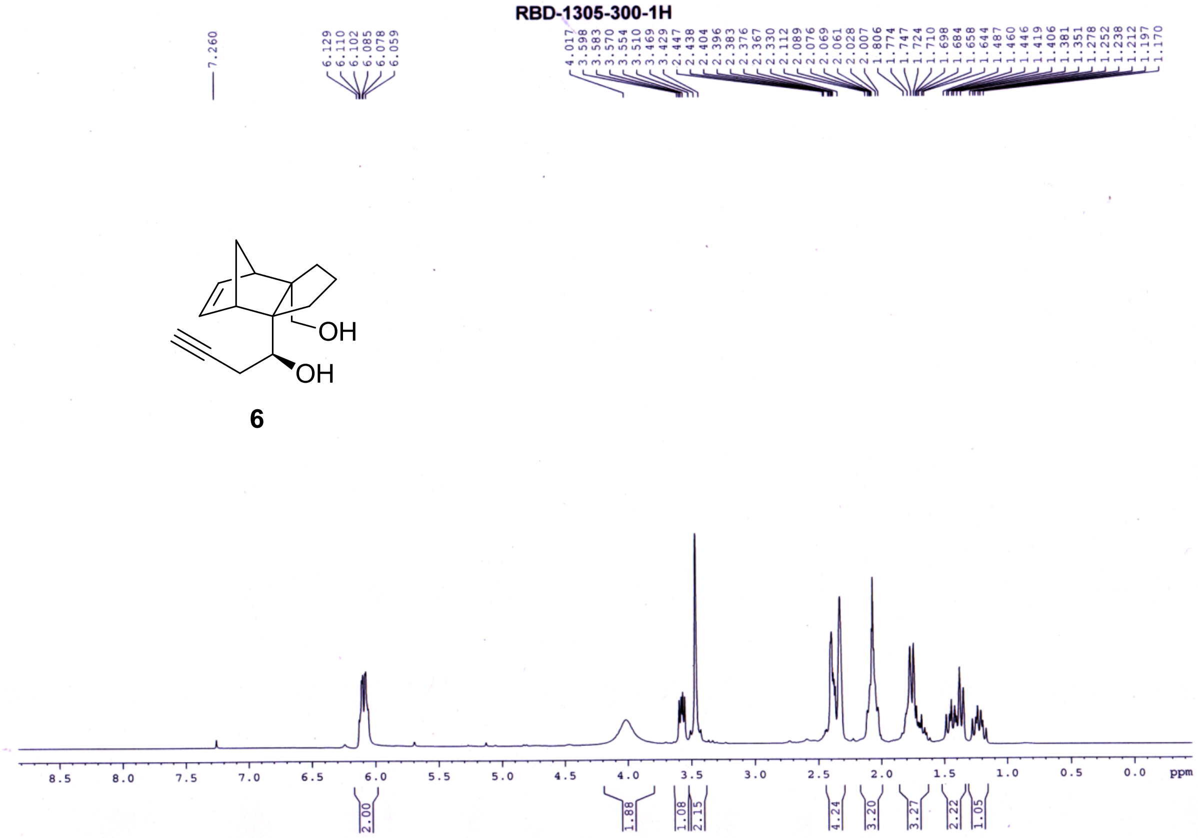
RBD-1305-300-1H
OH
OH
6
ppm

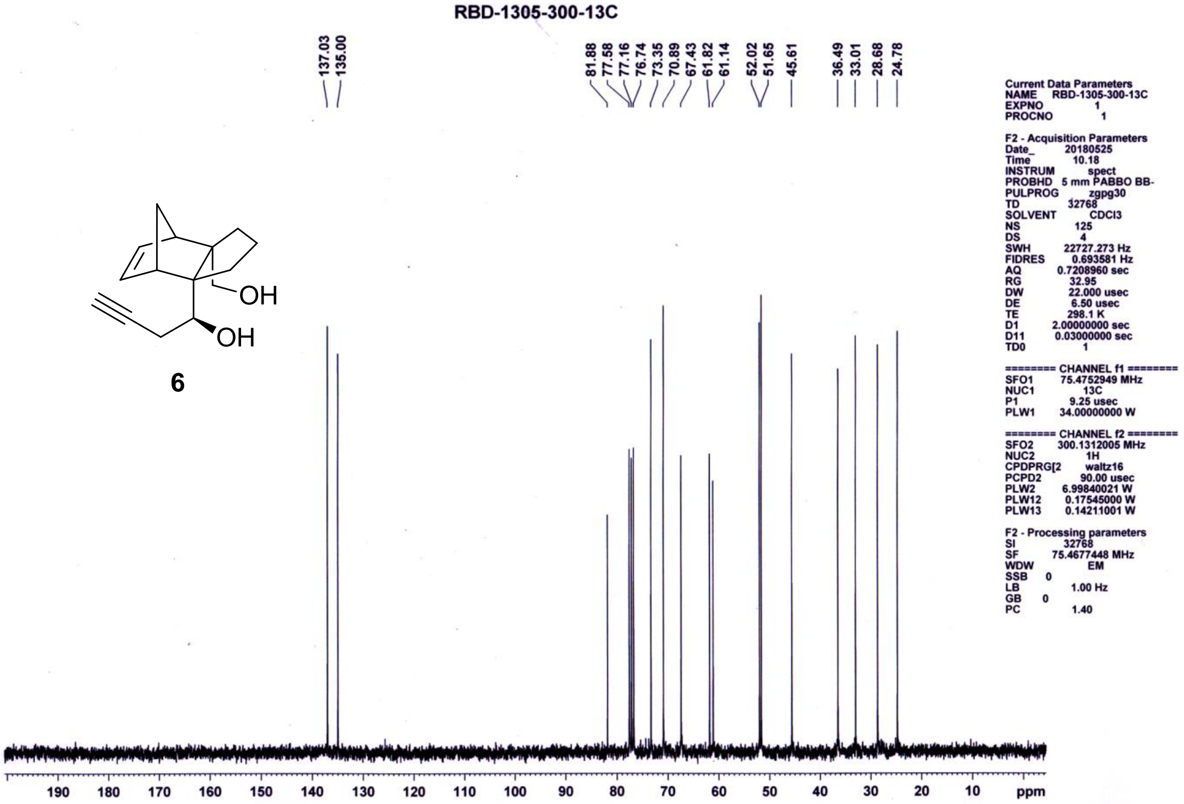
OH
OH
6
RBD-1305-300-13C
137.03
135.00
81.88
77.58
77.16
76.74
73.35
70.89
67.43
61.82
61.14
52.02
51.65
45.61
36.49
33.01
28.68
24.78
Current Data Parameters
NAME RBD-1305-300-13C
EXPNO 1
PROCNO 1
F2 - Acquisition Parameters
Date_ 20180525
Time 10.18
INSTRUM spect
PROBHD 5 mm PABBO BB-
PULPROG zgpg30
TD 32768
SOLVENT CDCl3
NS 125
DS 4
SWH 22727.273 Hz
FIDRES 0.693581 Hz
AQ 0.7208960 sec
RG 32.95
DW 22.000 usec
DE 6.50 usec
TE 298.1 K
D1 2.00000000 sec
D11 0.03000000 sec
TD0 1
======== CHANNEL f1 ========
SFO1 75.4752949 MHz
NUC1 13C
P1 9.25 usec
PLW1 34.00000000 W
======== CHANNEL f2 ========
SFO2 300.1312005 MHz
NUC2 1H
CPDPRG[2 waltz16
PCPD2 90.00 usec
PLW2 6.99840021 W
PLW12 0.17545000 W
PLW13 0.14211001 W
F2 - Processing parameters
SI 32768
SF 75.4677448 MHz
WDW EM
SSB 0
LB 1.00 Hz
GB 0
PC 1.40
190 180 170 160 150 140 130 120 110 100 90 80 70 60 50 40 30 20 10 ppm

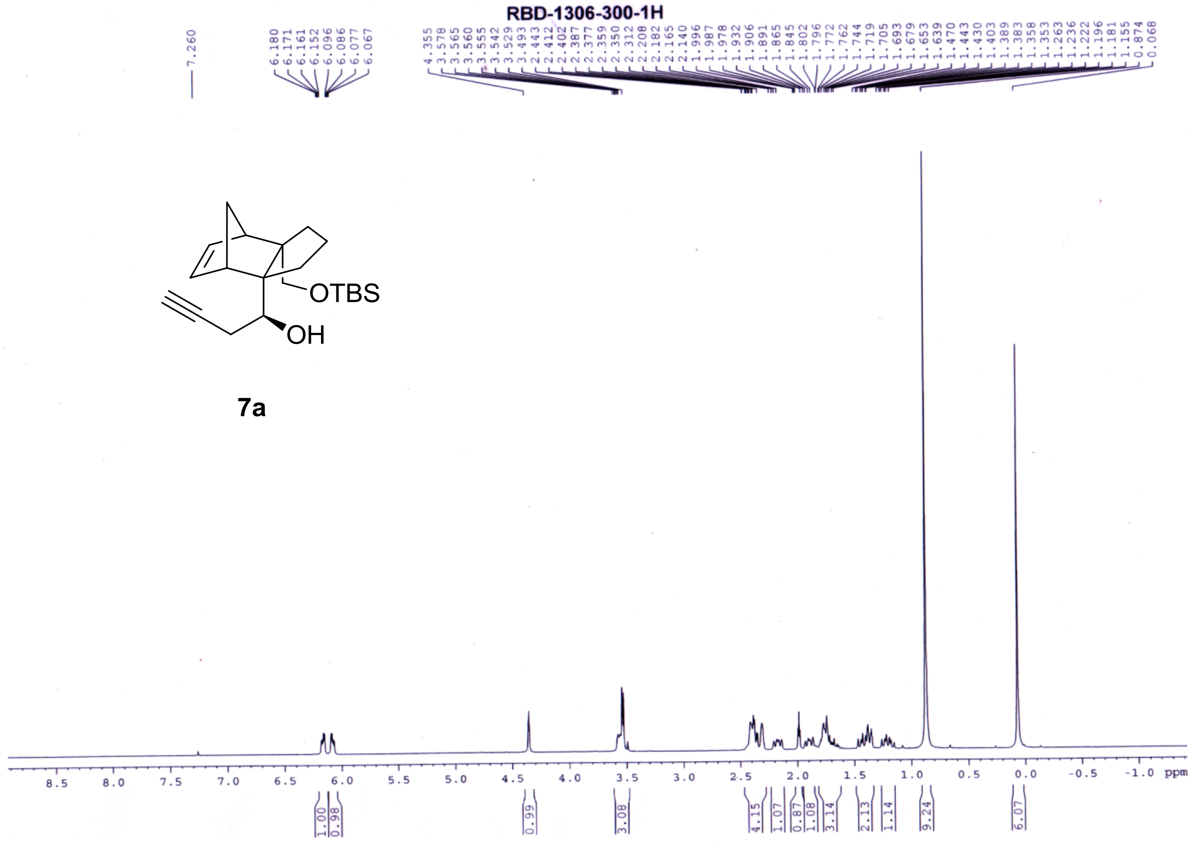
RBD-1306-300-1H
OTBS
OH
7a
ppm

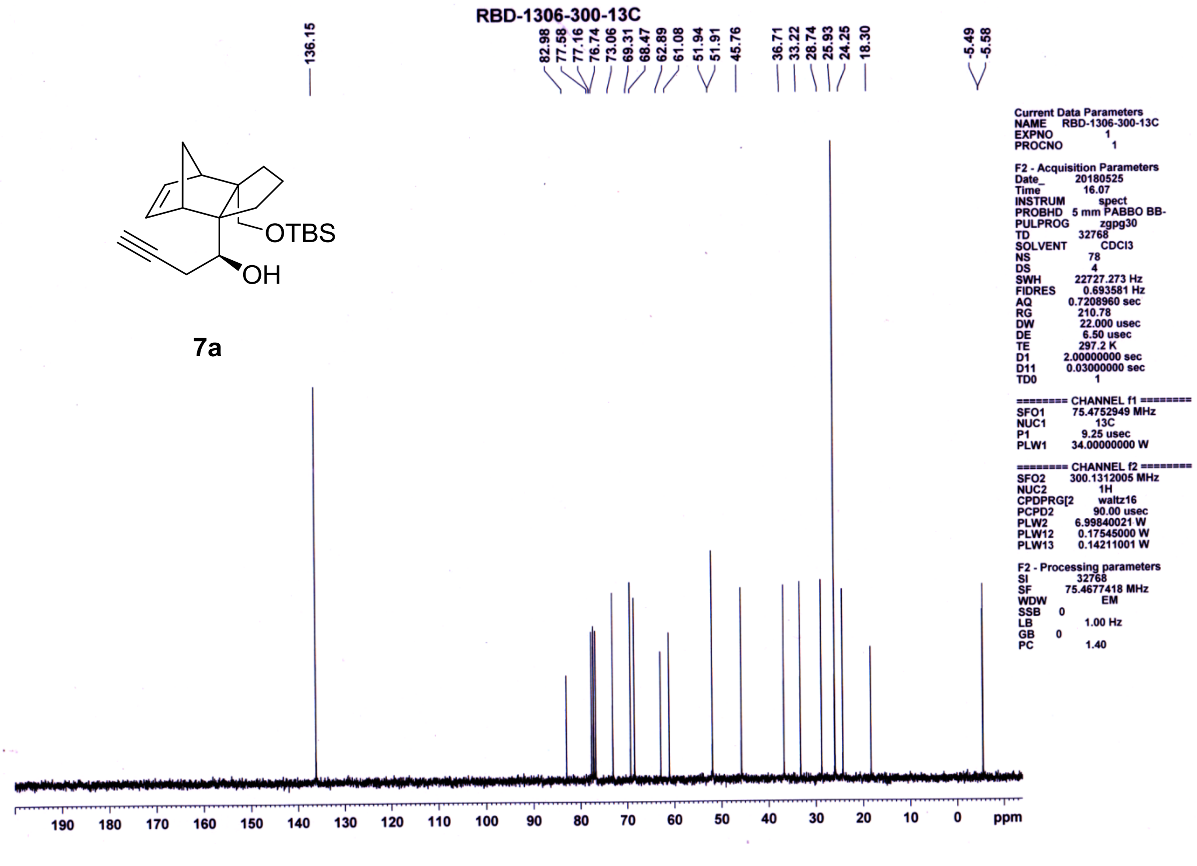
RBD-1306-300-13C
136.15
82.98
77.58
77.16
76.74
73.06
69.31
68.47
62.89
61.08
51.94
51.91
45.76
36.71
33.22
28.74
25.93
24.25
18.30
-5.49
-5.58
OTBS
OH
7a
ppm
Current Data Parameters
NAME RBD-1306-300-13C
EXPNO 1
PROCNO 1
F2 - Acquisition Parameters
Date_ 20180525
Time 16.07
INSTRUM spect
PROBHD 5 mm PABBO BB-
PULPROG zgpg30
TD 32768
SOLVENT CDCl3
NS 78
DS 4
SWH 22727.273 Hz
FIDRES 0.693581 Hz
AQ 0.7208960 sec
RG 210.78
DW 22.000 usec
DE 6.50 usec
TE 297.2 K
D1 2.00000000 sec
D11 0.03000000 sec
TD0 1
======== CHANNEL f1 ========
SFO1 75.4752949 MHz
NUC1 13C
P1 9.25 usec
PLW1 34.00000000 W
======== CHANNEL f2 ========
SFO2 300.1312005 MHz
NUC2 1H
CPDPRG[2 waltz16
PCPD2 90.00 usec
PLW2 6.99840021 W
PLW12 0.17545000 W
PLW13 0.14211001 W
F2 - Processing parameters
SI 32768
SF 75.4677418 MHz
WDW EM
SSB 0
LB 1.00 Hz
GB 0
PC 1.40

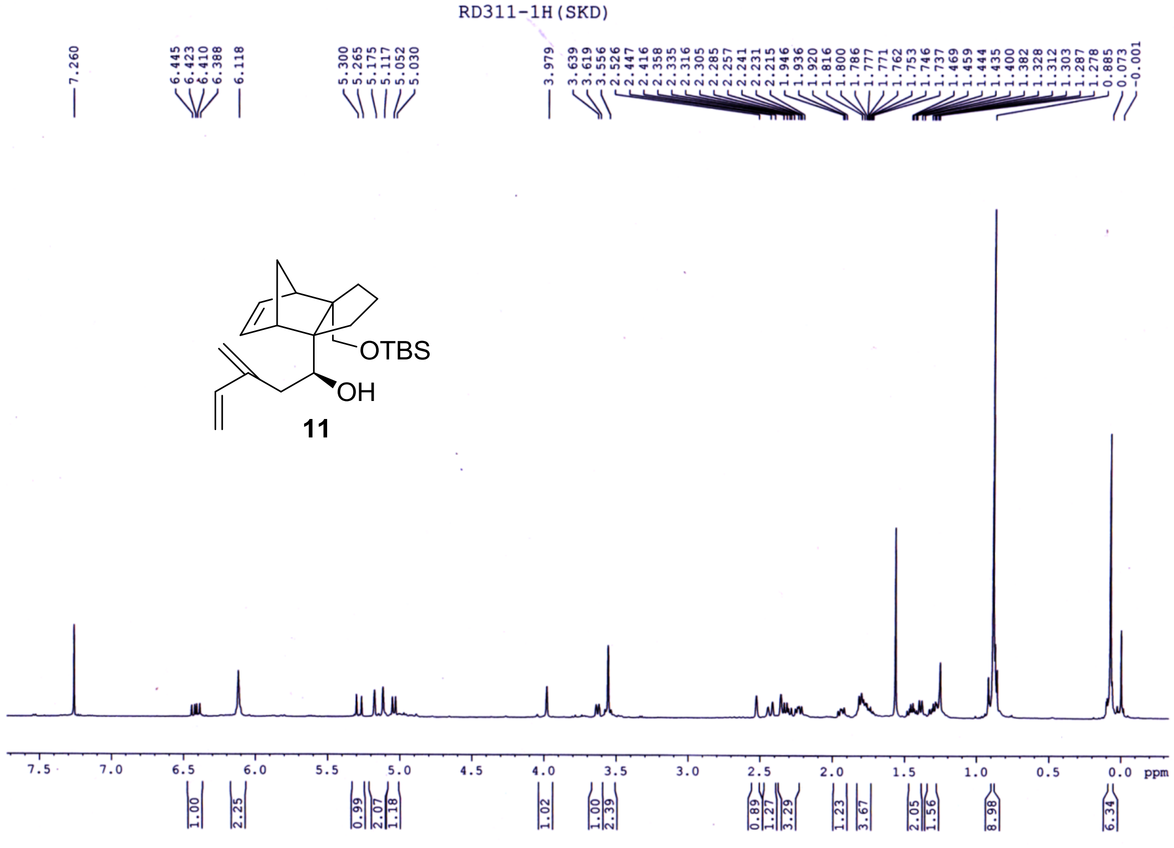
RD311-1H(SKD)
OTBS
OH
11
ppm

RB167.US-13C(SKD)

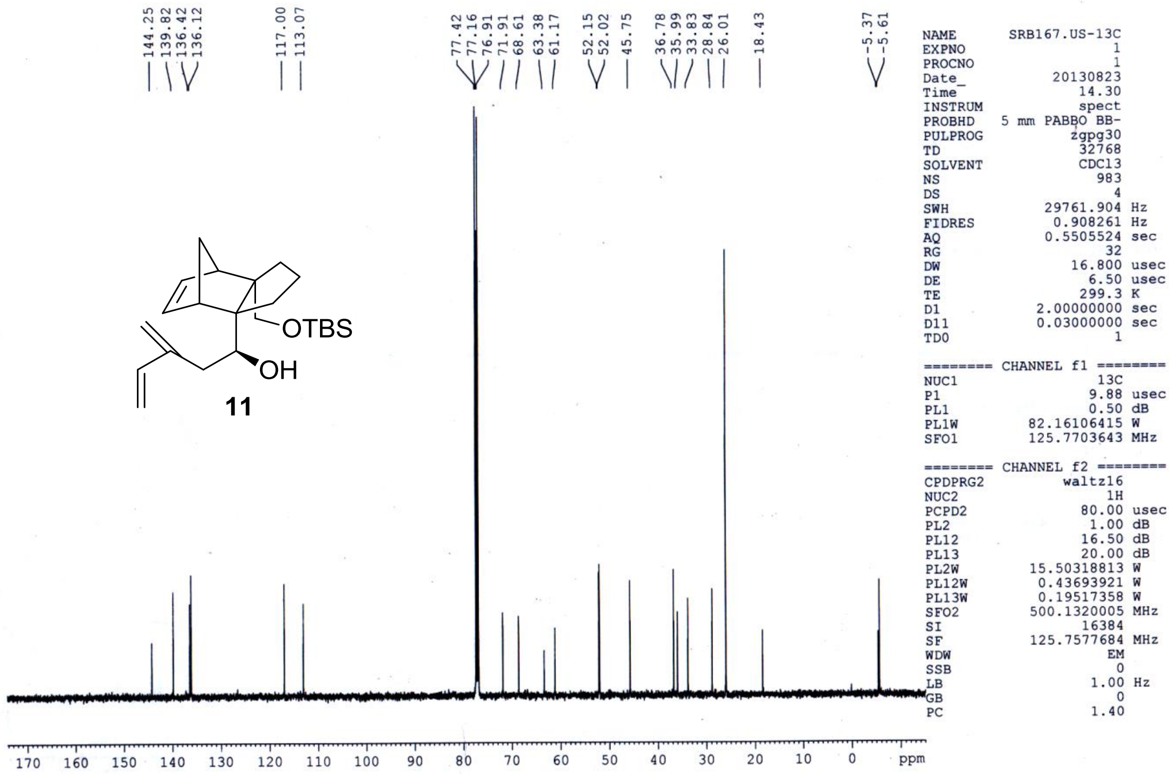

11

NAME	SRB167.US-13C
EXPNO	1
PROCNO	1
Date_	20130823
Time	14.30
INSTRUM	spect
PROBHD	5 mm PABBO BB-
PULPROG	zgpg30
TD	32768
SOLVENT	CDCl3
NS	983
DS	4
SWH	29761.904 Hz
FIDRES	0.908261 Hz
AQ	0.5505524 sec
RG	32
DW	16.800 usec
DE	6.50 usec
TE	299.3 K
D1	2.00000000 sec
D11	0.03000000 sec
TD0	1

======== CHANNEL f1 ========

NUC1	13C
P1	9.88 usec
PL1	0.50 dB
PL1W	82.16106415 W
SFO1	125.7703643 MHz

======== CHANNEL f2 ========

CPDPRG2	waltz16
NUC2	1H
PCPD2	80.00 usec
PL2	1.00 dB
PL12	16.50 dB
PL13	20.00 dB
PL2W	15.50318813 W
PL12W	0.43693921 W
PL13W	0.19517358 W
SFO2	500.1320005 MHz
SI	16384
SF	125.7577684 MHz
WDW	EM
SSB	0
LB	1.00 Hz
GB	0
PC	1.40

RD-312.1-300-1H

13

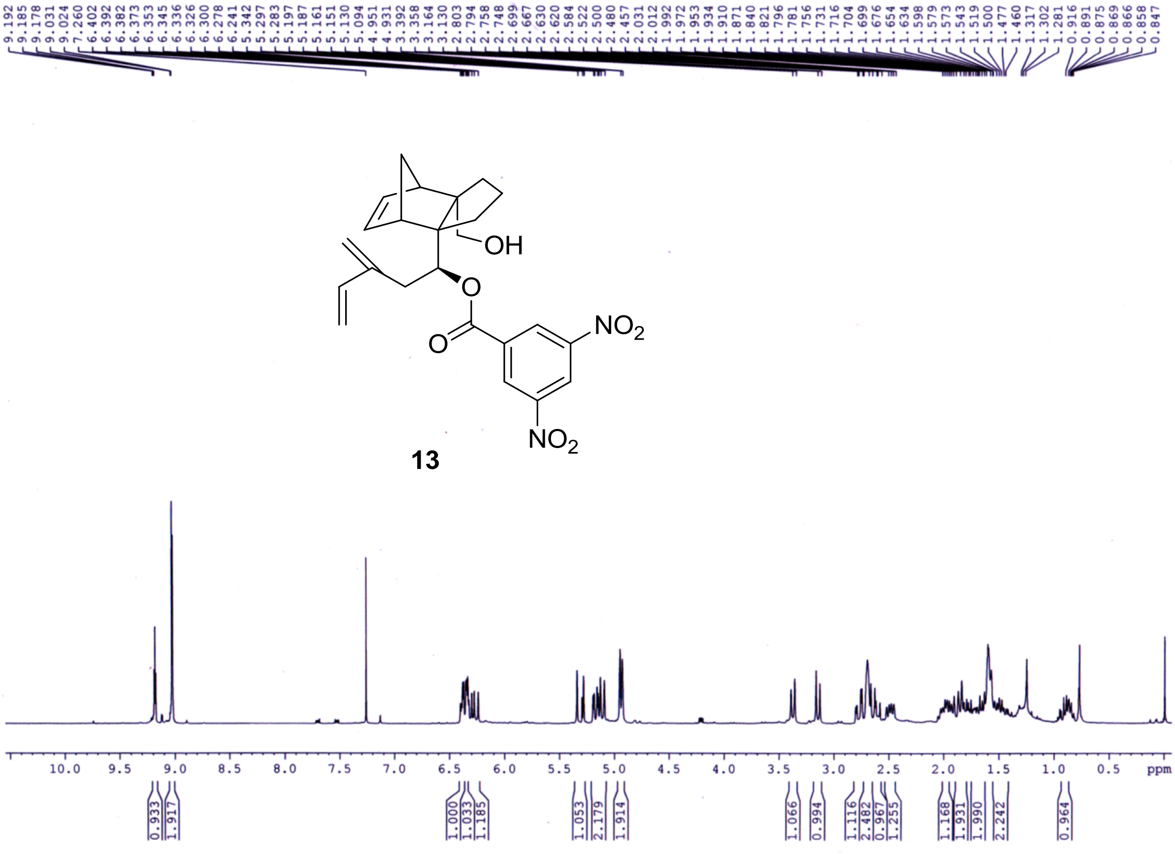

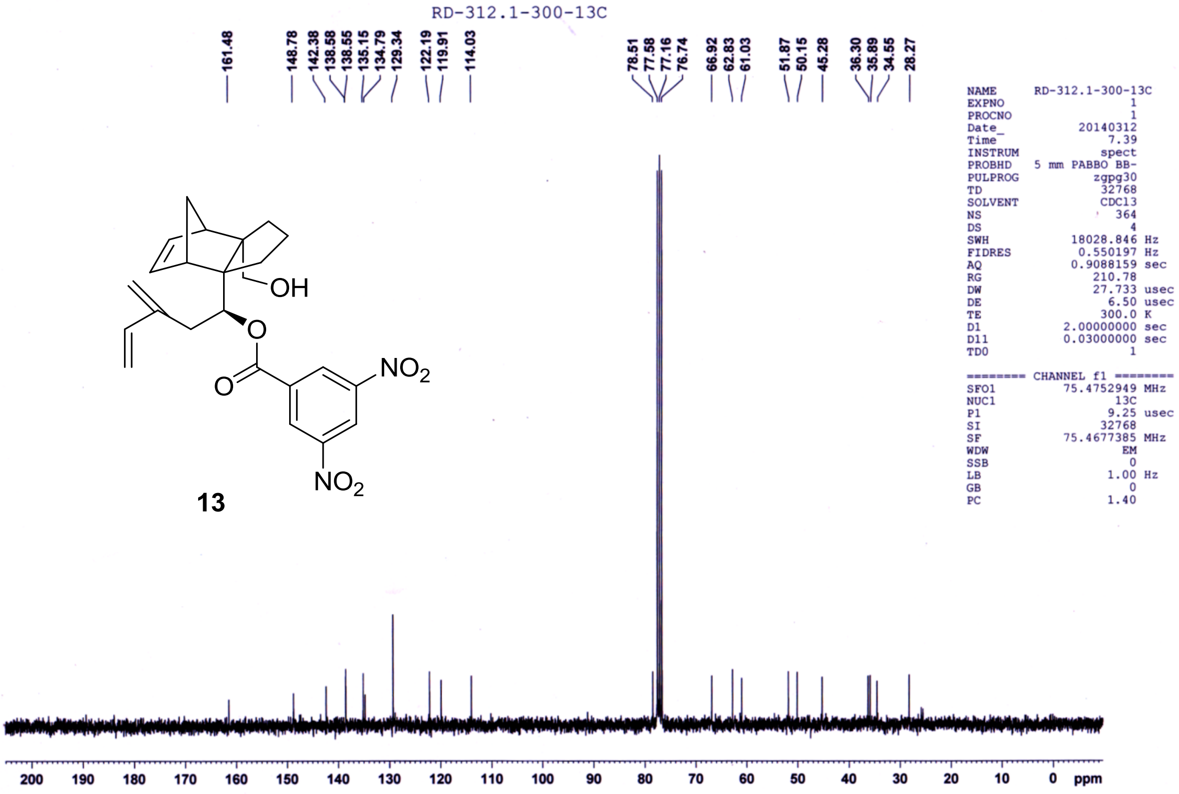
RD-312.1-300-13C
161.48
148.78
142.38
138.58
138.55
135.15
134.79
129.34
122.19
119.91
114.03
78.51
77.58
77.16
76.74
66.92
62.83
61.03
51.87
50.15
45.28
36.30
35.89
34.55
28.27
OH
O
O
NO2
NO2
13
NAME RD-312.1-300-13C
EXPNO 1
PROCNO 1
Date_ 20140312
Time 7.39
INSTRUM spect
PROBHD 5 mm PABBO BB-
PULPROG zgpg30
TD 32768
SOLVENT CDCl3
NS 364
DS 4
SWH 18028.846 Hz
FIDRES 0.550197 Hz
AQ 0.9088159 sec
RG 210.78
DW 27.733 usec
DE 6.50 usec
TE 300.0 K
D1 2.00000000 sec
D11 0.03000000 sec
TD0 1
======== CHANNEL f1 ========
SFO1 75.4752949 MHz
NUC1 13C
P1 9.25 usec
SI 32768
SF 75.4677385 MHz
WDW EM
SSB 0
LB 1.00 Hz
GB 0
PC 1.40
200 190 180 170 160 150 140 130 120 110 100 90 80 70 60 50 40 30 20 10 0 ppm

RD-222-1H(SR) 500MHz

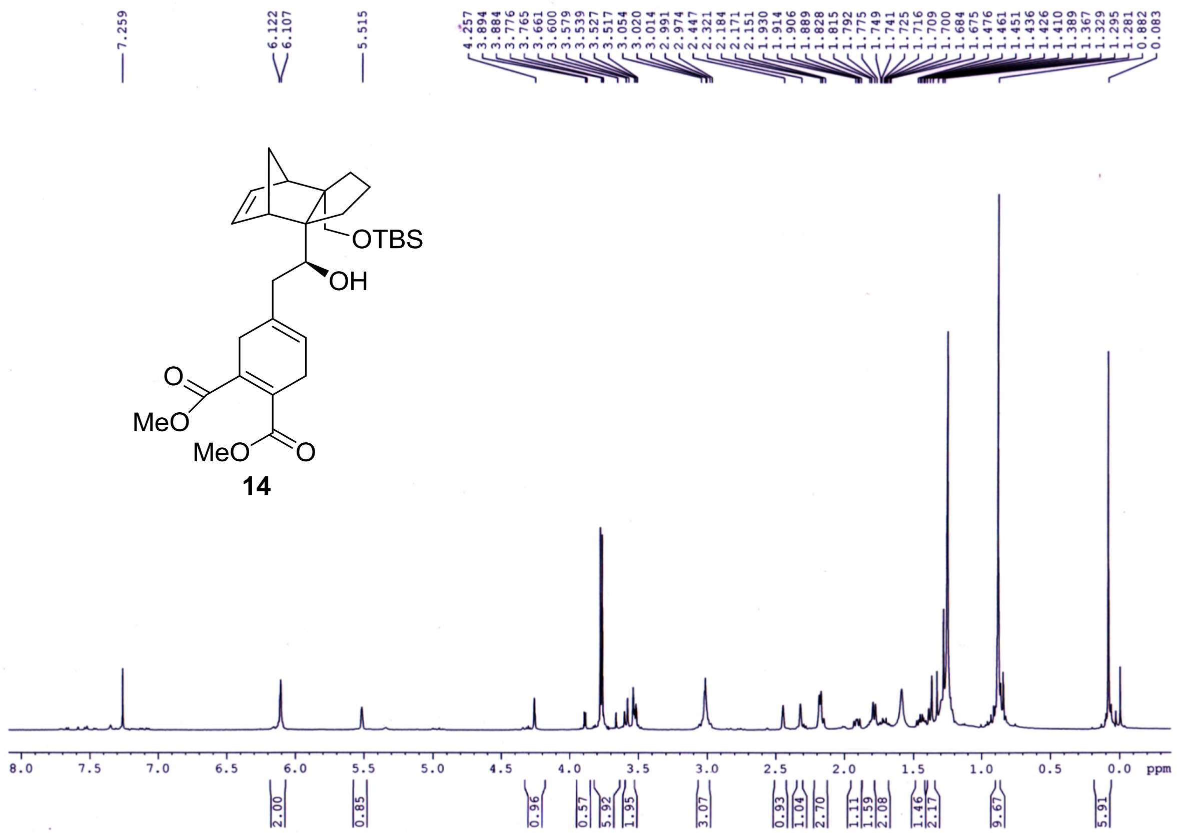

RB-194A-13C(SR)

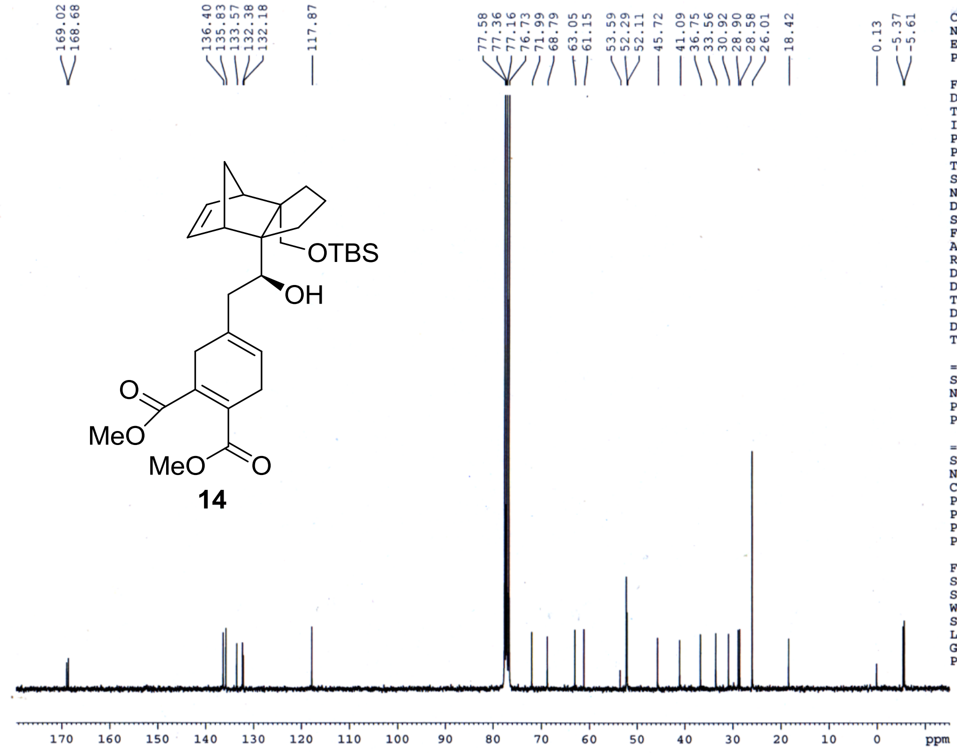

Current Data Parameters
NAME SRB-194A-13C
EXPNO 1
PROCNO 1

F2 - Acquisition Parameters
Date_ 20130913
Time 9.18
INSTRUM spect
PROBHD 5 mm PABBO BB-
PULPROG zgpg30
TD 32768
SOLVENT CDCl3
NS 17092
DS 4
SWH 21929.824 Hz
FIDRES 0.669245 Hz
AQ 0.7471104 sec
RG 210.78
DW 22.800 usec
DE 6.50 usec
TE 298.5 K
D1 2.00000000 sec
D11 0.03000000 sec
TD0 1

======== CHANNEL f1 ========
SFO1 75.4752949 MHz
NUC1 13C
P1 9.25 usec
PLW1 34.00000000 W

======== CHANNEL f2 ========
SFO2 300.1312005 MHz
NUC2 1H
CPDPRG[2 waltz16
PCPD2 90.00 usec
PLW2 6.99840021 W
PLW12 0.17545000 W
PLW13 0.14211001 W

F2 - Processing parameters
SI 32768
SF 75.4677375 MHz
WDW EM
SSB 0
LB 1.00 Hz
GB 0
PC 1.40

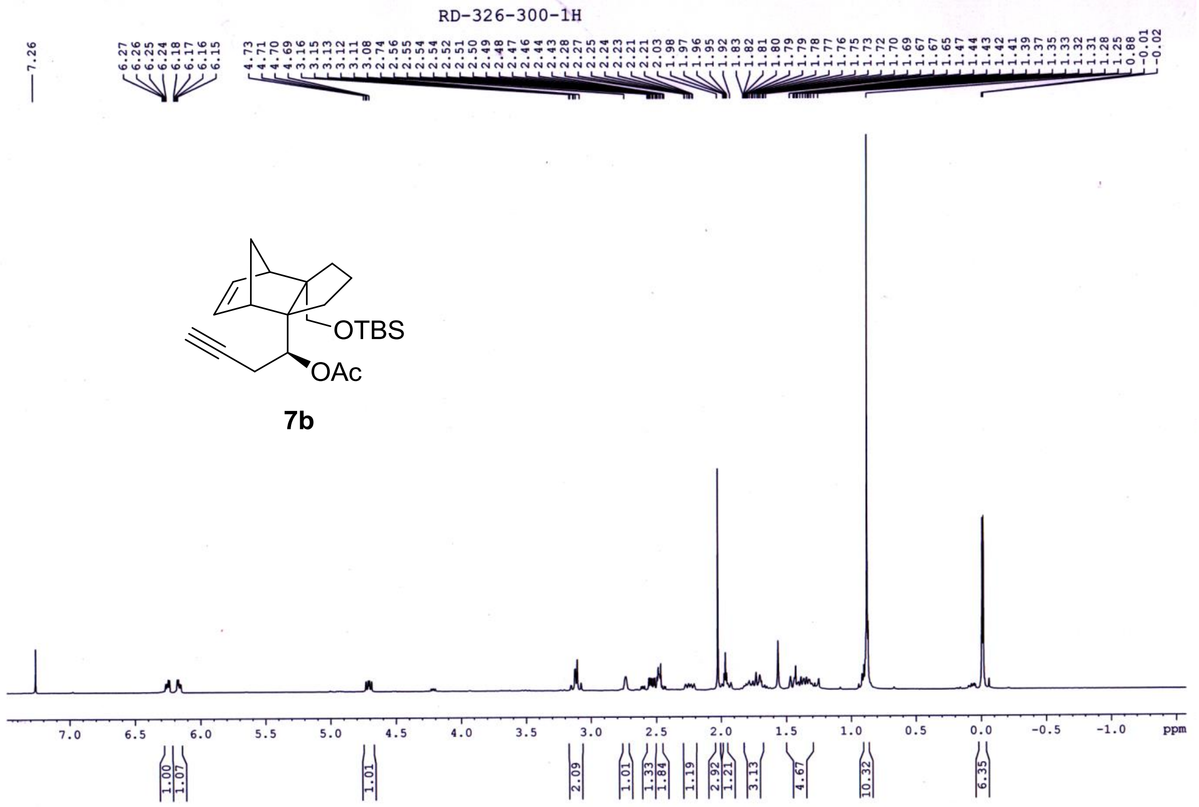
RD-326-300-1H
7.26
6.27 6.26 6.25 6.24 6.18 6.17 6.16 6.15
4.73 4.71 4.70 4.69 3.16 3.15 3.13 3.12 3.11 3.08 2.74 2.56 2.55 2.54 2.54 2.52 2.51 2.50 2.49 2.48 2.47 2.46 2.44 2.43 2.28 2.27 2.25 2.24 2.23 2.21 2.21 2.03 1.98 1.97 1.96 1.95 1.92 1.83 1.82 1.81 1.80 1.79 1.79 1.78 1.77 1.76 1.75 1.73 1.72 1.70 1.69 1.67 1.65 1.47 1.44 1.43 1.42 1.41 1.39 1.37 1.35 1.33 1.32 1.31 1.28 1.25 0.88 -0.01 -0.02
OTBS
OAc
7b
7.0 6.5 6.0 5.5 5.0 4.5 4.0 3.5 3.0 2.5 2.0 1.5 1.0 0.5 0.0 -0.5 -1.0 ppm
1.00 1.07 1.01 2.09 1.01 1.33 1.84 1.19 2.92 1.21 3.13 4.67 10.32 6.35

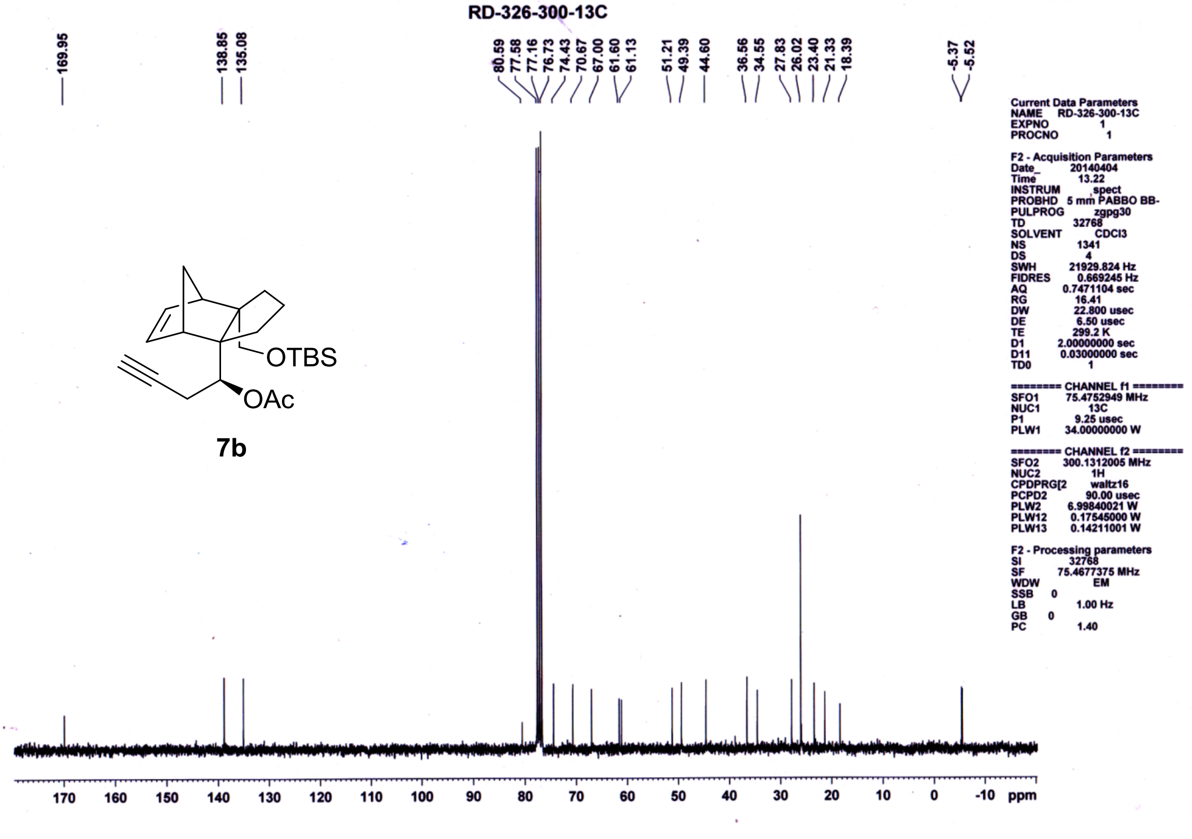
RD-326-300-13C
169.95
138.85 135.08
80.59 77.58 77.16 76.73 74.43 70.67 67.00 61.60 61.13
51.21 49.39 44.60
36.56 34.55 27.83 26.02 23.40 21.33 18.39
-5.37 -5.52
OTBS
OAc
7b
Current Data Parameters
NAME RD-326-300-13C
EXPNO 1
PROCNO 1
F2 - Acquisition Parameters
Date_ 20140404
Time 13.22
INSTRUM spect
PROBHD 5 mm PABBO BB-
PULPROG zgpg30
TD 32768
SOLVENT CDCl3
NS 1341
DS 4
SWH 21929.824 Hz
FIDRES 0.669245 Hz
AQ 0.7471104 sec
RG 16.41
DW 22.800 usec
DE 6.50 usec
TE 299.2 K
D1 2.00000000 sec
D11 0.03000000 sec
TD0 1
======== CHANNEL f1 ========
SFO1 75.4752949 MHz
NUC1 13C
P1 9.25 usec
PLW1 34.00000000 W
======== CHANNEL f2 ========
SFO2 300.1312005 MHz
NUC2 1H
CPDPRG[2 waltz16
PCPD2 90.00 usec
PLW2 6.99840021 W
PLW12 0.17545000 W
PLW13 0.14211001 W
F2 - Processing parameters
SI 32768
SF 75.4677375 MHz
WDW EM
SSB 0
LB 1.00 Hz
GB 0
PC 1.40
170 160 150 140 130 120 110 100 90 80 70 60 50 40 30 20 10 0 -10 ppm

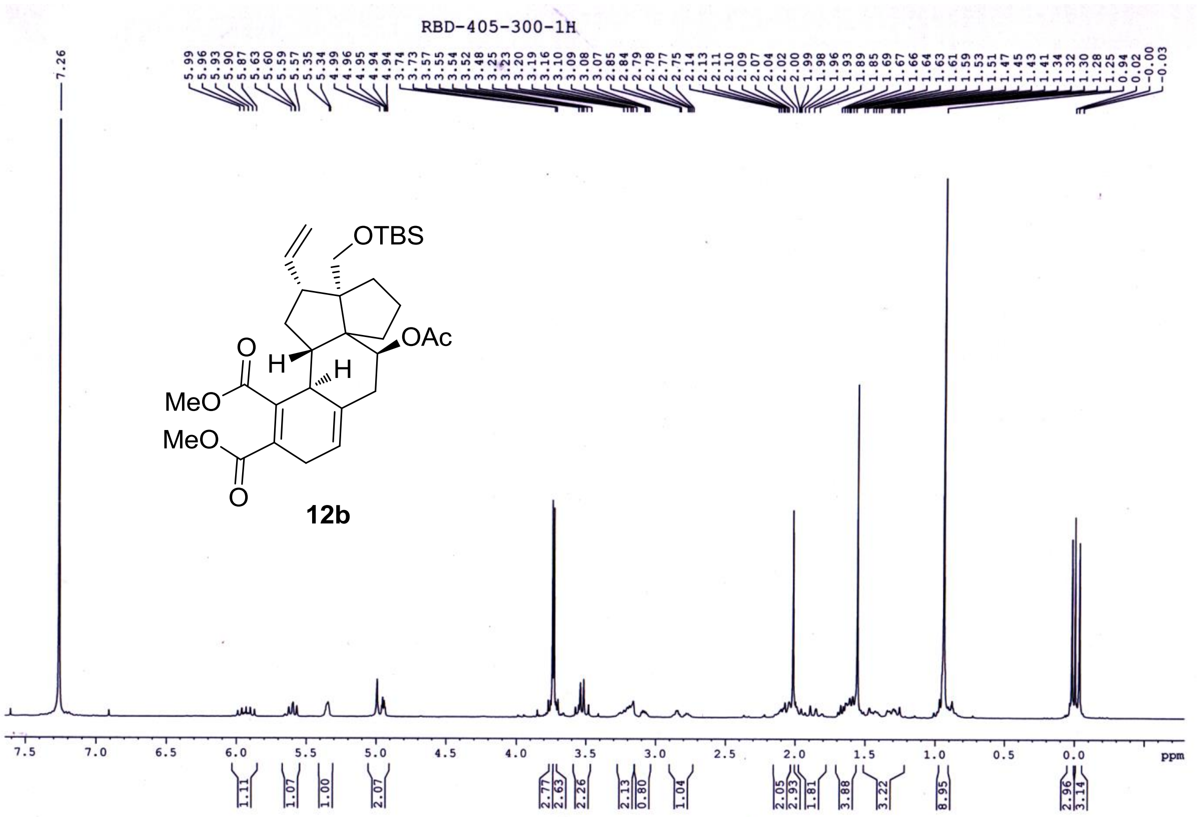
RBD-405-300-1H
OTBS
OAc
H
H
MeO
MeO
12b
ppm

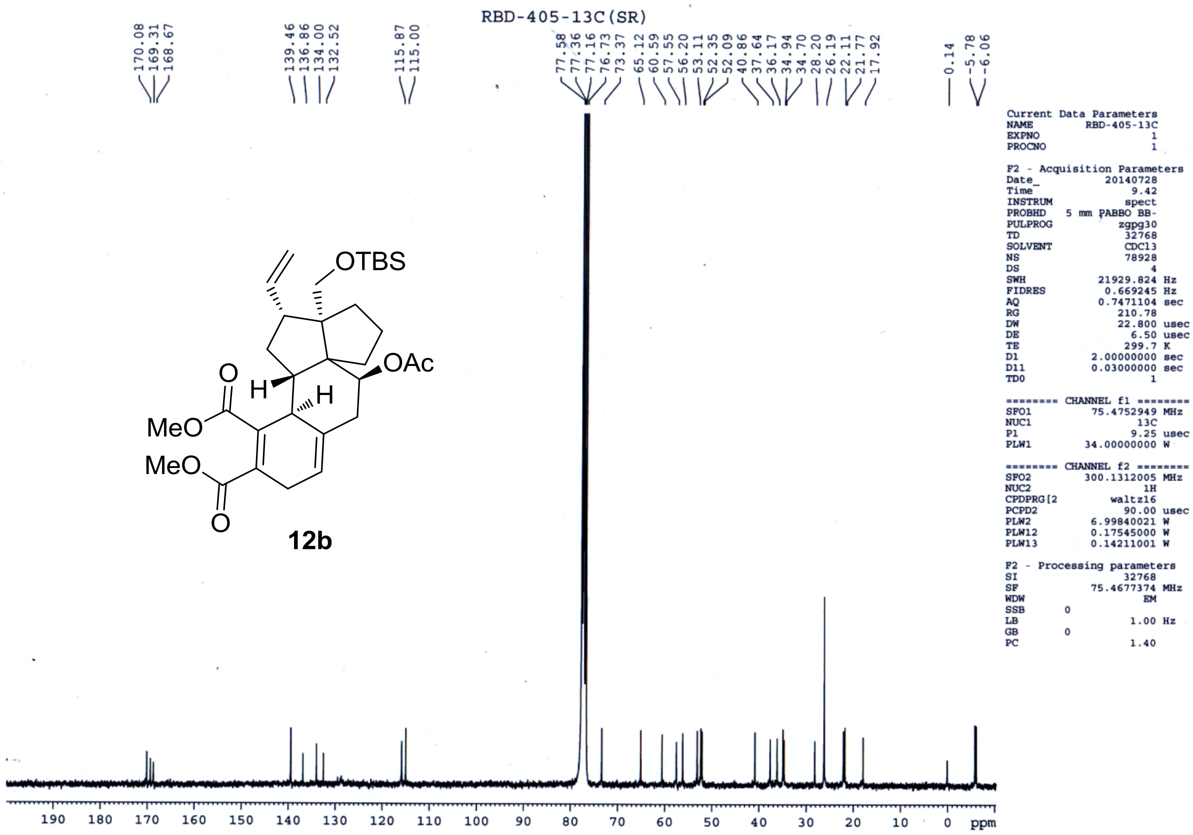
RBD-405-13C(SR)
170.08
169.31
168.67
139.46
136.86
134.00
132.52
115.87
115.00
77.58
77.36
77.16
76.73
73.37
65.12
60.59
57.55
56.20
53.11
52.35
52.09
40.86
37.64
36.17
34.94
34.70
28.20
26.19
22.11
21.77
17.92
0.14
-5.78
-6.06
OTBS
OAc
H
H
MeO
MeO
O
O
12b
Current Data Parameters
NAME RBD-405-13C
EXPNO 1
PROCNO 1
F2 - Acquisition Parameters
Date_ 20140728
Time 9.42
INSTRUM spect
PROBHD 5 mm PABBO BB-
PULPROG zgpg30
TD 32768
SOLVENT CDCl3
NS 78928
DS 4
SWH 21929.824 Hz
FIDRES 0.669245 Hz
AQ 0.7471104 sec
RG 210.78
DW 22.800 usec
DE 6.50 usec
TE 299.7 K
D1 2.00000000 sec
D11 0.03000000 sec
TD0 1
======== CHANNEL f1 ========
SFO1 75.4752949 MHz
NUC1 13C
P1 9.25 usec
PLW1 34.00000000 W
======== CHANNEL f2 ========
SFO2 300.1312005 MHz
NUC2 1H
CPDPRG[2 waltz16
PCPD2 90.00 usec
PLW2 6.99840021 W
PLW12 0.17545000 W
PLW13 0.14211001 W
F2 - Processing parameters
SI 32768
SF 75.4677374 MHz
WDW EM
SSB 0
LB 1.00 Hz
GB 0
PC 1.40
190 180 170 160 150 140 130 120 110 100 90 80 70 60 50 40 30 20 10 0 ppm

RBD-1303-300-1H

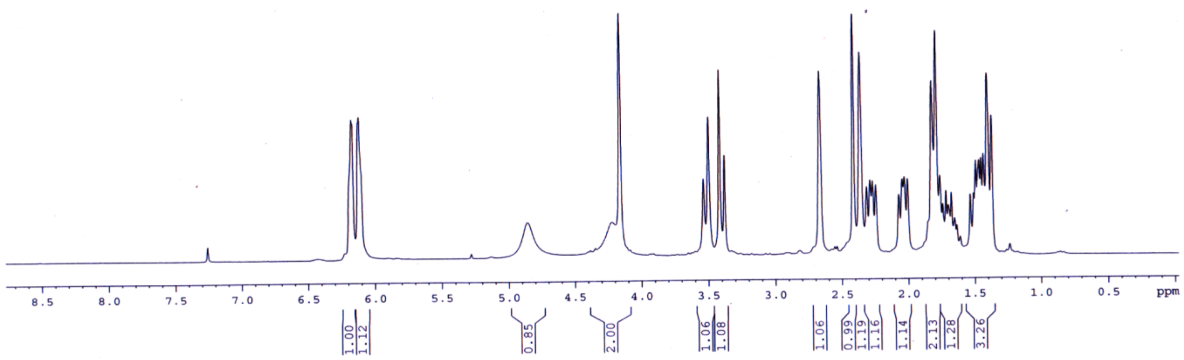

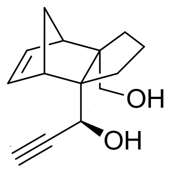

15

RBD-1303-300-13C

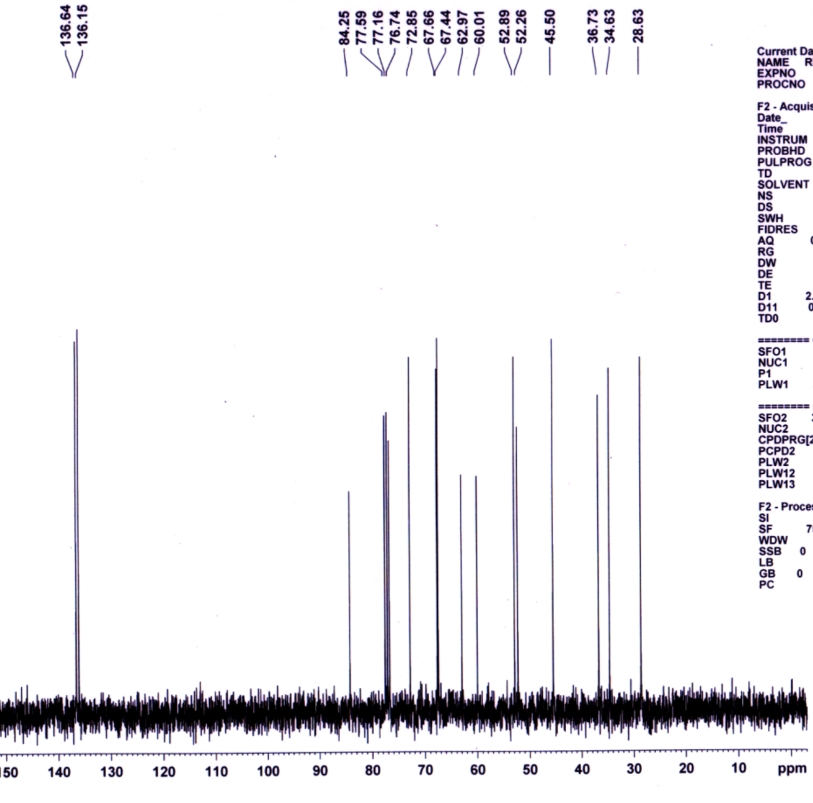

Current Data Parameters
NAME RBD-1303-300-13C
EXPNO 1
PROCNO 1

F2 - Acquisition Parameters
Date_ 20180518
Time 10.06
INSTRUM spect
PROBHD 5 mm PABBO BB-
PULPROG zgpg30
TD 32768
SOLVENT CDCl3
NS 20
DS 2
SWH 21929.824 Hz
FIDRES 0.669245 Hz
AQ 0.7471104 sec
RG 32.95
DW 22.800 usec
DE 6.50 usec
TE 296.6 K
D1 2.00000000 sec
D11 0.03000000 sec
TD0 1

======== CHANNEL f1 ========
SFO1 75.4752949 MHz
NUC1 13C
P1 9.25 usec
PLW1 34.00000000 W

======== CHANNEL f2 ========
SFO2 300.1312005 MHz
NUC2 1H
CPDPRG[2 waltz16
PCPD2 90.00 usec
PLW2 6.99840021 W
PLW12 0.17545000 W
PLW13 0.14211001 W

F2 - Processing parameters
SI 16384
SF 75.4677420 MHz
WDW EM
SSB 0
LB 1.00 Hz
GB 0
PC 1.40

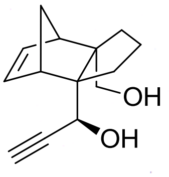

15

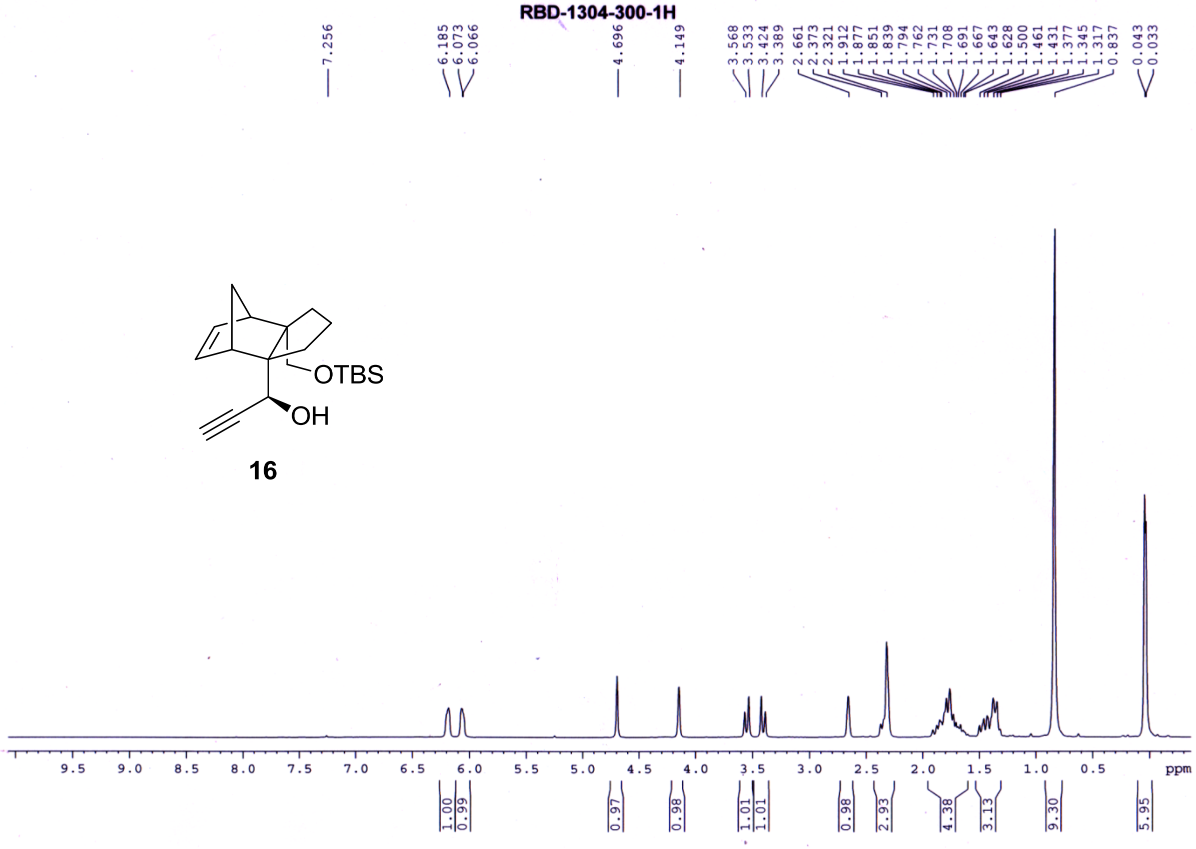

16

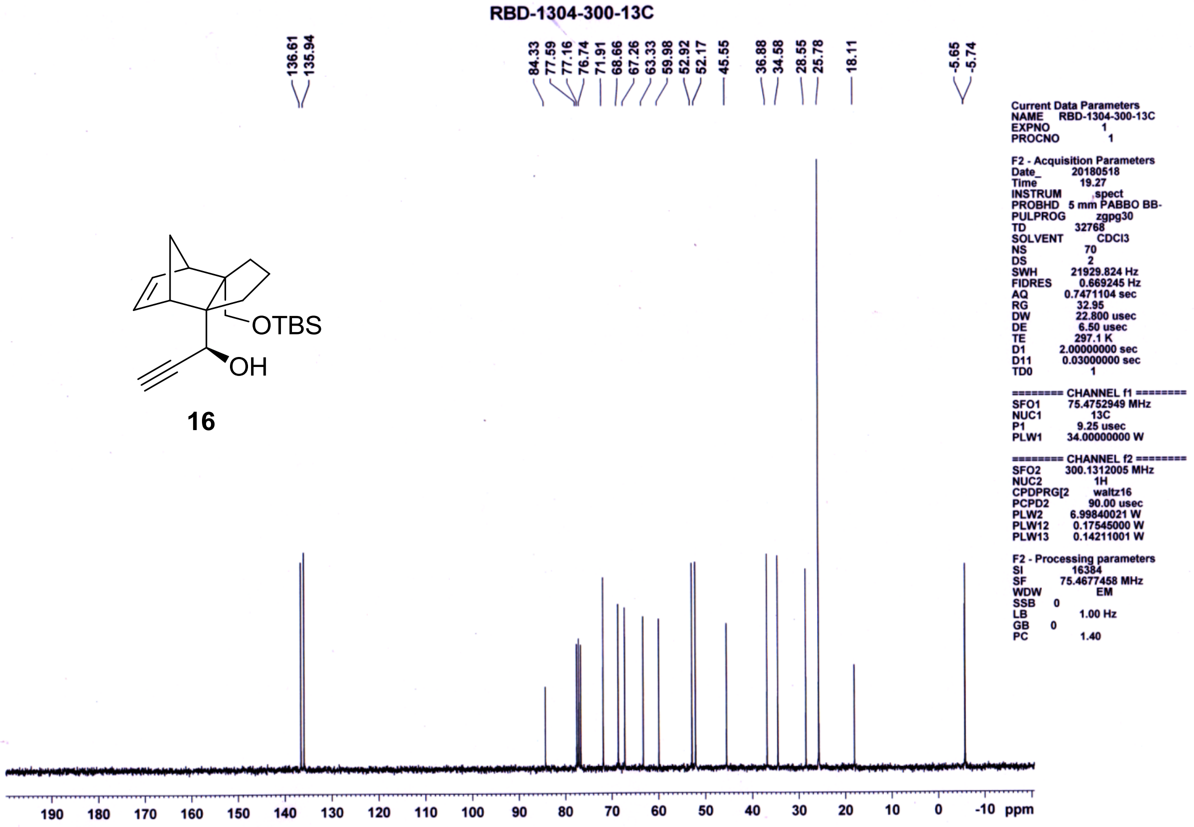
RBD-1304-300-13C
136.61
135.94
84.33
77.59
77.16
76.74
71.91
68.66
67.26
63.33
59.98
52.92
52.17
45.55
36.88
34.58
28.55
25.78
18.11
-5.65
-5.74
OTBS
OH
16
Current Data Parameters
NAME RBD-1304-300-13C
EXPNO 1
PROCNO 1
F2 - Acquisition Parameters
Date_ 20180518
Time 19.27
INSTRUM spect
PROBHD 5 mm PABBO BB-
PULPROG zgpg30
TD 32768
SOLVENT CDCl3
NS 70
DS 2
SWH 21929.824 Hz
FIDRES 0.669245 Hz
AQ 0.7471104 sec
RG 32.95
DW 22.800 usec
DE 6.50 usec
TE 297.1 K
D1 2.00000000 sec
D11 0.03000000 sec
TD0 1
======== CHANNEL f1 ========
SFO1 75.4752949 MHz
NUC1 13C
P1 9.25 usec
PLW1 34.00000000 W
======== CHANNEL f2 ========
SFO2 300.1312005 MHz
NUC2 1H
CPDPRG[2 waltz16
PCPD2 90.00 usec
PLW2 6.99840021 W
PLW12 0.17545000 W
PLW13 0.14211001 W
F2 - Processing parameters
SI 16384
SF 75.4677458 MHz
WDW EM
SSB 0
LB 1.00 Hz
GB 0
PC 1.40
190 180 170 160 150 140 130 120 110 100 90 80 70 60 50 40 30 20 10 0 -10 ppm

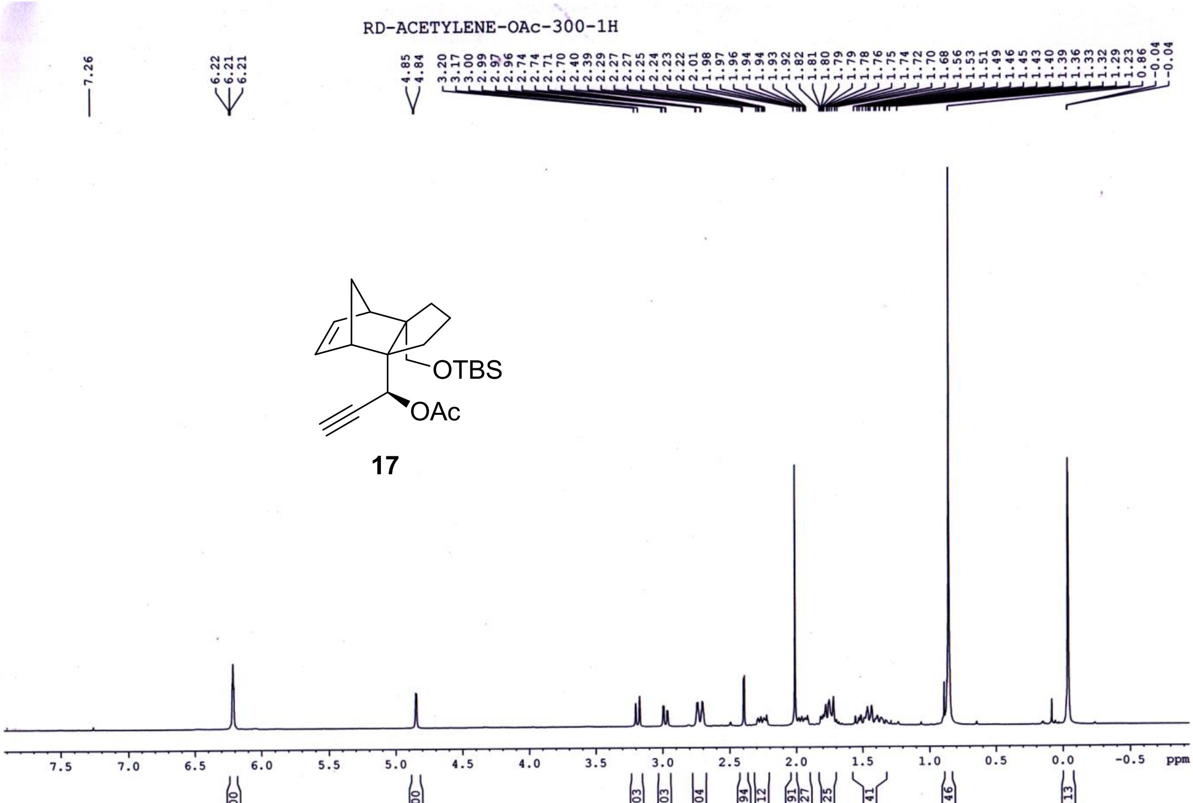
RD-ACETYLENE-OAc-300-1H
7.26
6.22
6.21
6.21
4.85
4.84
3.20
3.17
3.00
2.99
2.97
2.96
2.74
2.74
2.71
2.70
2.40
2.39
2.29
2.27
2.27
2.25
2.24
2.23
2.22
2.01
1.98
1.97
1.96
1.94
1.94
1.93
1.92
1.82
1.81
1.80
1.79
1.79
1.78
1.76
1.75
1.74
1.72
1.70
1.68
1.56
1.53
1.51
1.49
1.46
1.45
1.43
1.40
1.39
1.36
1.33
1.32
1.29
1.23
0.86
-0.04
-0.04
OTBS
OAc
17
7.5 7.0 6.5 6.0 5.5 5.0 4.5 4.0 3.5 3.0 2.5 2.0 1.5 1.0 0.5 0.0 -0.5 ppm
2.00
1.00
1.03
1.03
2.04
0.94
1.12
2.91
1.27
3.25
3.41
9.46
6.13

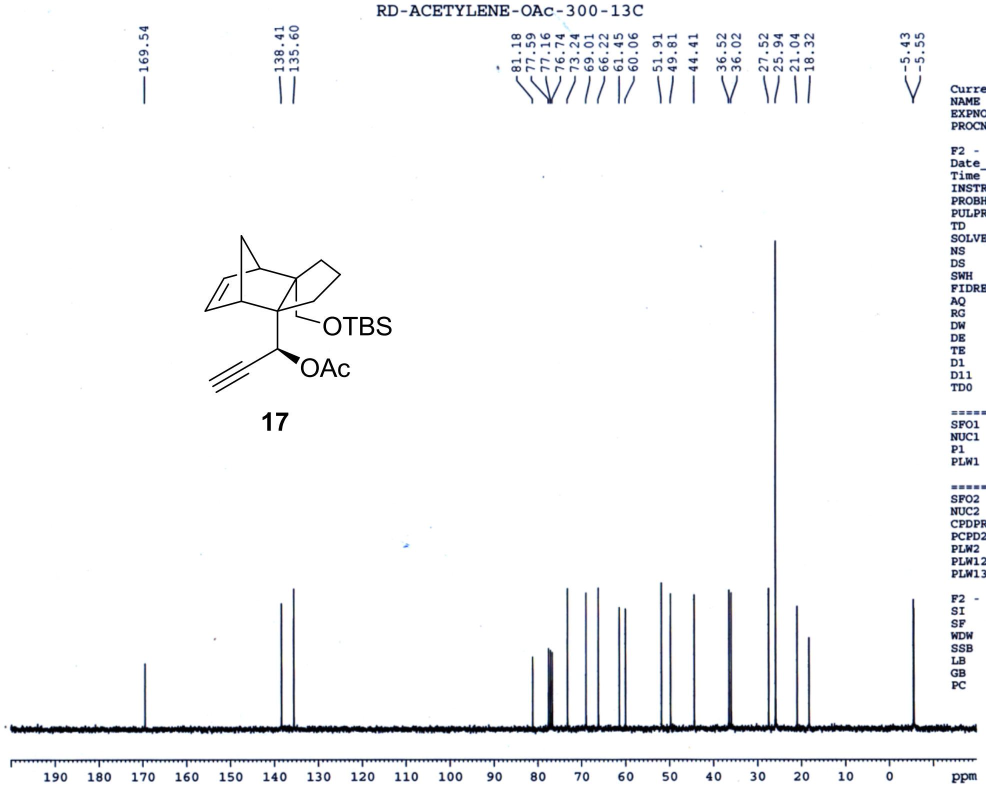


```
Current Data Parameters
NAME      RD-ACETYLENE-OAc-300-13C
EXPNO                    1
PROCNO                   1

F2 - Acquisition Parameters
Date_             20140409
Time                 10.07
INSTRUM              spect
PROBHD    5 mm PABBO BB-
PULPROG            zgpg30
TD                   32768
SOLVENT              CDCl3
NS                      69
DS                       4
SWH              21929.824 Hz
FIDRES            0.669245 Hz
AQ               0.7471104 sec
RG                   16.41
DW                  22.800 usec
DE                    6.50 usec
TE                   298.6 K
D1              2.00000000 sec
D11             0.03000000 sec
TD0                      1

======== CHANNEL f1 ========
SFO1            75.4752949 MHz
NUC1                   13C
P1                    9.25 usec
PLW1           34.00000000 W

======== CHANNEL f2 ========
SFO2           300.1312005 MHz
NUC2                    1H
CPDPRG[2           waltz16
PCPD2                90.00 usec
PLW2            6.99840021 W
PLW12           0.17545000 W
PLW13           0.14211001 W

F2 - Processing parameters
SI                   32768
SF              75.4677408 MHz
WDW                     EM
SSB       0
LB                    1.00 Hz
GB        0
PC                    1.40
```

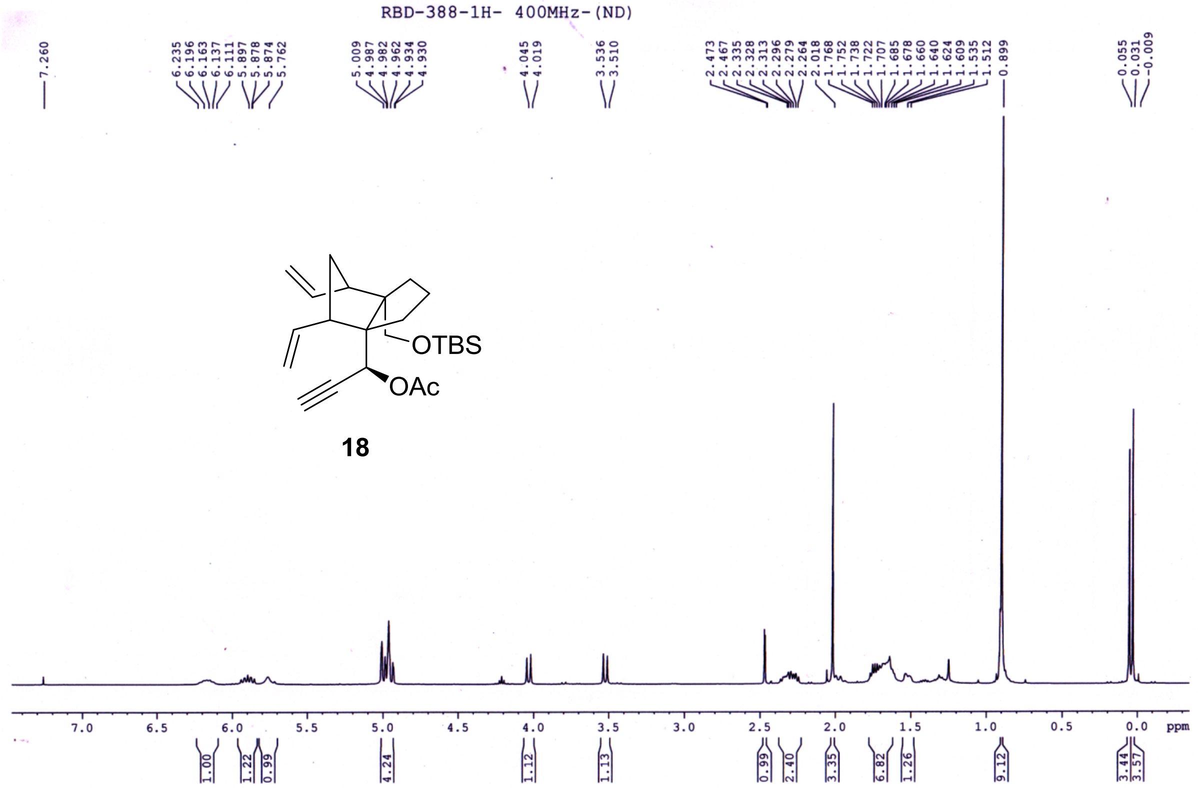
RBD-388-1H- 400MHz-(ND)
7.260
6.235 6.196 6.163 6.137 6.111 5.897 5.878 5.874 5.762
5.009 4.987 4.982 4.962 4.934 4.930
4.045 4.019
3.536 3.510
2.473 2.467 2.335 2.328 2.313 2.296 2.279 2.264 2.018 1.768 1.752 1.738 1.722 1.707 1.685 1.678 1.660 1.640 1.624 1.609 1.535 1.512 0.899
0.055 0.031 -0.009
OTBS
OAc
18
ppm
1.00 1.22 0.99 4.24 1.12 1.13 0.99 2.40 3.35 6.82 1.26 9.12 3.44 3.57

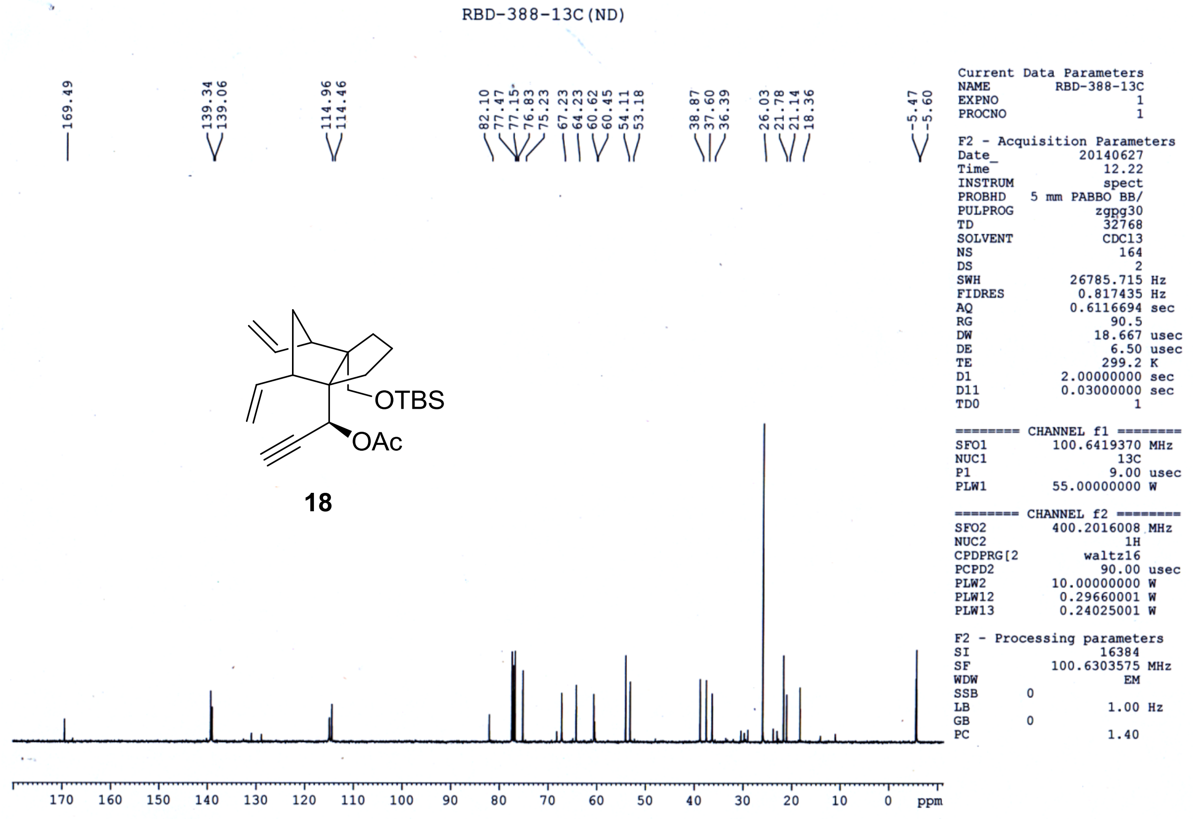
RBD-388-13C(ND)
169.49
139.34 139.06
114.96 114.46
82.10 77.47 77.15 76.83 75.23 67.23 64.23 60.62 60.45 54.11 53.18
38.87 37.60 36.39
26.03 21.78 21.14 18.36
-5.47 -5.60
OTBS
OAc
18
ppm
Current Data Parameters
NAME RBD-388-13C
EXPNO 1
PROCNO 1
F2 - Acquisition Parameters
Date_ 20140627
Time 12.22
INSTRUM spect
PROBHD 5 mm PABBO BB/
PULPROG zgpg30
TD 32768
SOLVENT CDCl3
NS 164
DS 2
SWH 26785.715 Hz
FIDRES 0.817435 Hz
AQ 0.6116694 sec
RG 90.5
DW 18.667 usec
DE 6.50 usec
TE 299.2 K
D1 2.00000000 sec
D11 0.03000000 sec
TD0 1
======== CHANNEL f1 ========
SFO1 100.6419370 MHz
NUC1 13C
P1 9.00 usec
PLW1 55.00000000 W
======== CHANNEL f2 ========
SFO2 400.2016008 MHz
NUC2 1H
CPDPRG[2 waltz16
PCPD2 90.00 usec
PLW2 10.00000000 W
PLW12 0.29660001 W
PLW13 0.24025001 W
F2 - Processing parameters
SI 16384
SF 100.6303575 MHz
WDW EM
SSB 0
LB 1.00 Hz
GB 0
PC 1.40

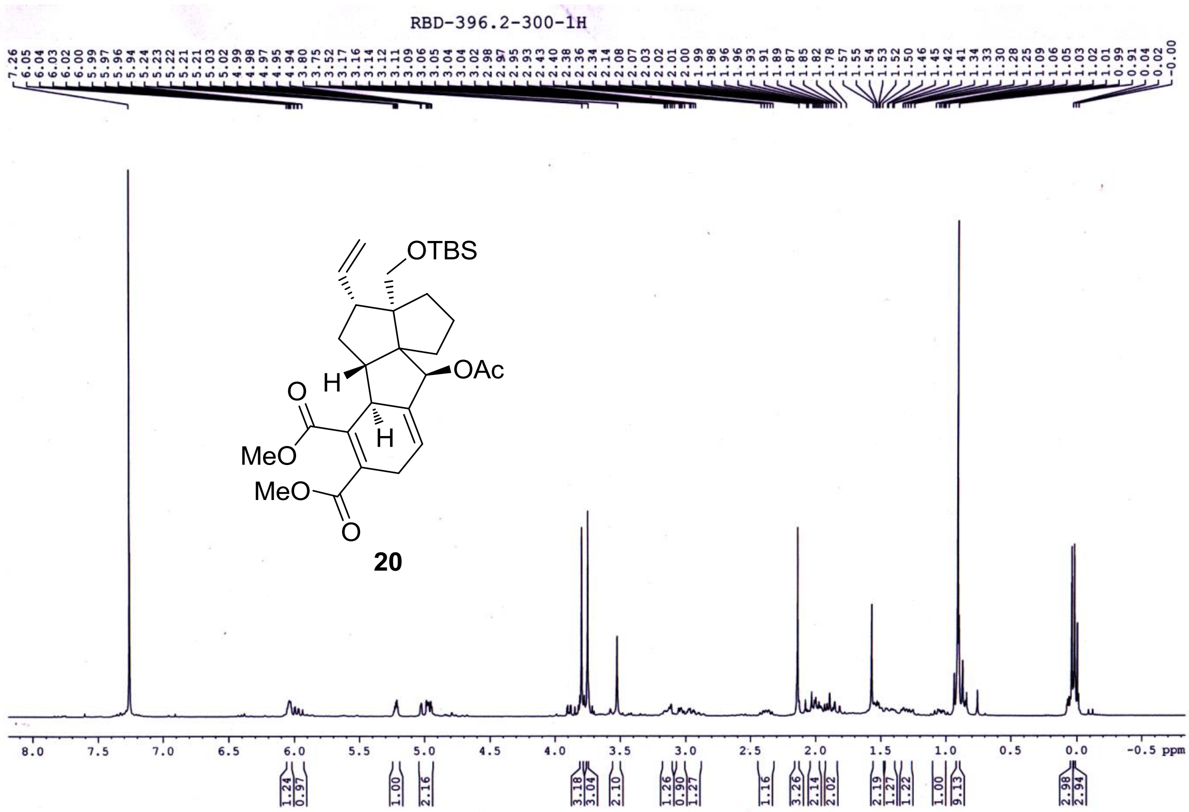

RBD-396.2-300-1H
7.26 6.05 6.04 6.03 6.02 6.00 5.99 5.97 5.96 5.94 5.24 5.23 5.22 5.21 5.03 5.02 4.99 4.98 4.97 4.95 4.94 3.80 3.75 3.52 3.17 3.16 3.14 3.12 3.11 3.09 3.06 3.05 3.04 3.02 2.98 2.97 2.95 2.93 2.43 2.40 2.38 2.36 2.34 2.14 2.08 2.07 2.03 2.02 2.01 2.00 1.99 1.98 1.96 1.93 1.91 1.89 1.87 1.85 1.82 1.78 1.57 1.55 1.54 1.53 1.52 1.50 1.46 1.45 1.42 1.41 1.34 1.33 1.30 1.28 1.25 1.09 1.06 1.05 1.03 1.02 1.01 0.99 0.91 0.04 0.02 -0.00
OTBS
OAc
H
H
O
MeO
MeO
O
20
8.0 7.5 7.0 6.5 6.0 5.5 5.0 4.5 4.0 3.5 3.0 2.5 2.0 1.5 1.0 0.5 0.0 -0.5 ppm
1.24 0.97 1.00 2.16 3.18 3.04 2.10 1.26 0.90 1.27 1.16 3.26 2.14 2.02 2.19 1.27 1.22 1.00 9.13 2.98 2.94

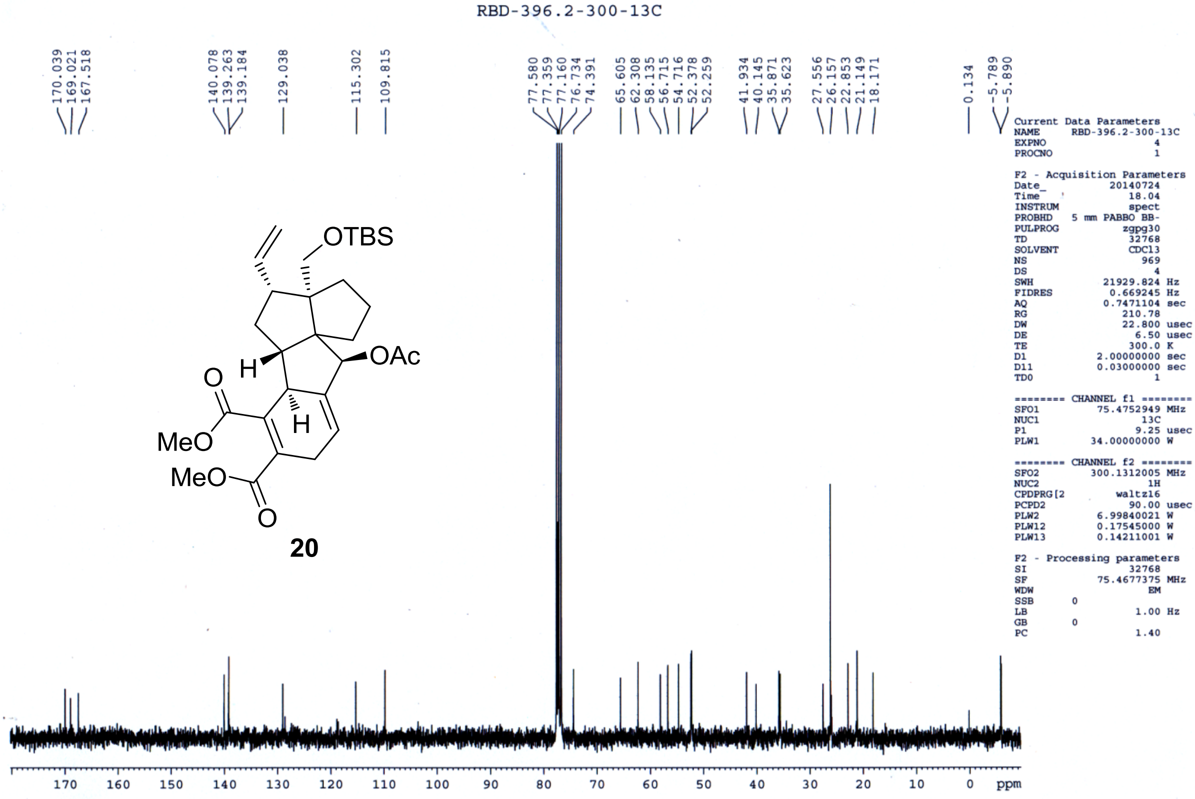

RBD-396.2-300-13C
170.039 169.021 167.518 140.078 139.263 139.184 129.038 115.302 109.815 77.580 77.359 77.160 76.734 74.391 65.605 62.308 58.135 56.715 54.716 52.378 52.259 41.934 40.145 35.871 35.623 27.556 26.157 22.853 21.149 18.171 0.134 -5.789 -5.890
OTBS
OAc
H
H
O
MeO
MeO
O
20
Current Data Parameters
NAME RBD-396.2-300-13C
EXPNO 4
PROCNO 1
F2 - Acquisition Parameters
Date_ 20140724
Time 18.04
INSTRUM spect
PROBHD 5 mm PABBO BB-
PULPROG zgpg30
TD 32768
SOLVENT CDCl3
NS 969
DS 4
SWH 21929.824 Hz
FIDRES 0.669245 Hz
AQ 0.7471104 sec
RG 210.78
DW 22.800 usec
DE 6.50 usec
TE 300.0 K
D1 2.00000000 sec
D11 0.03000000 sec
TD0 1
======== CHANNEL f1 ========
SFO1 75.4752949 MHz
NUC1 13C
P1 9.25 usec
PLW1 34.00000000 W
======== CHANNEL f2 ========
SFO2 300.1312005 MHz
NUC2 1H
CPDPRG[2 waltz16
PCPD2 90.00 usec
PLW2 6.99840021 W
PLW12 0.17545000 W
PLW13 0.14211001 W
F2 - Processing parameters
SI 32768
SF 75.4677375 MHz
WDW EM
SSB 0
LB 1.00 Hz
GB 0
PC 1.40
170 160 150 140 130 120 110 100 90 80 70 60 50 40 30 20 10 0 ppm
